# Dosage regulation, and variation in gene expression and copy number of human Y chromosome ampliconic genes

**DOI:** 10.1371/journal.pgen.1008369

**Published:** 2019-09-16

**Authors:** Rahulsimham Vegesna, Marta Tomaszkiewicz, Paul Medvedev, Kateryna D. Makova

**Affiliations:** 1 Bioinformatics and Genomics Graduate Program, The Huck Institutes for the Life Sciences, Pennsylvania State University, University Park, PA, United States of America; 2 Department of Biochemistry and Molecular Biology, Pennsylvania State University, University Park, PA, United States of America; 3 Department of Biology, Pennsylvania State University, University Park, PA, United States of America; 4 Department of Computer Science and Engineering, Pennsylvania State University, University Park, PA, United States of America; 5 Center for Computational Biology and Bioinformatics, Pennsylvania State University, University Park, PA, United States of America; 6 Center for Medical Genomics, Pennsylvania State University, University Park, PA, United States of America; Cornell University, UNITED STATES

## Abstract

The Y chromosome harbors nine multi-copy ampliconic gene families expressed exclusively in testis. The gene copies within each family are >99% identical to each other, which poses a major challenge in evaluating their copy number. Recent studies demonstrated high variation in Y ampliconic gene copy number among humans. However, how this variation affects expression levels in human testis remains understudied. Here we developed a novel computational tool Ampliconic Copy Number Estimator (AmpliCoNE) that utilizes read sequencing depth information to estimate Y ampliconic gene copy number per family. We applied this tool to whole-genome sequencing data of 149 men with matched testis expression data whose samples are part of the Genotype-Tissue Expression (GTEx) project. We found that the Y ampliconic gene families with low copy number in humans were deleted or pseudogenized in non-human great apes, suggesting relaxation of functional constraints. Among the Y ampliconic gene families, higher copy number leads to higher expression. Within the Y ampliconic gene families, copy number does not influence gene expression, rather a high tolerance for variation in gene expression was observed in testis of presumably healthy men. No differences in gene expression levels were found among major Y haplogroups. Age positively correlated with expression levels of the *HSFY* and *PRY* gene families in the African subhaplogroup E1b, but not in the European subhaplogroups R1b and I1. We also found that expression of five Y ampliconic gene families is coordinated with that of their non-Y (i.e. X or autosomal) homologs. Indeed, five ampliconic gene families had consistently lower expression levels when compared to their non-Y homologs suggesting dosage regulation, while the *HSFY* family had higher expression levels than its X homolog and thus lacked dosage regulation.

## Author summary

The human genome harbors two sex chromosomes—X and Y. Among them, the Y chromosome is present only in males. Deletions of portions of this chromosome have been linked to male infertility, however exactly why the loss of these genes leads to this condition is not well understood. Here we study a group of Y chromosome genes called ampliconic genes, which are expressed in testis and are frequently deleted in males with infertility. These genes are organized in nine gene families, each of which harbors multiple copies of genes highly similar in sequence. In this study, we aimed to establish a baseline of their variation in copy number and in gene expression—one measure of genes’ functional output—by studying 149 healthy men. We found that testis tolerates a wide range of copy number and expression variation of Y ampliconic genes. Additionally, we demonstrated that gene expression within most Y ampliconic gene families depends on the expression levels of gene family members located outside of the Y chromosome, i.e. they undergo dosage regulation.

## Introduction

The human Y chromosome harbors 10.2 million bases (Mb) of ampliconic regions containing nine protein-coding multi-copy gene families [1]. These genes are important not only because of their association with male infertility [1,2] but also because they might hold the key to understanding the evolutionary forces that have shaped the Y chromosome. Ampliconic gene families show a high level of copy number variability [3–5] and, possibly, a similar variability in gene expression levels. Understanding the relationship between these two variabilities is an important step in the study of these genes. Yet, there has been no comprehensive investigation to-date that explores expression of these gene families and its connection to copy number at a large, population-level scale.

Studying ampliconic gene families has been a considerable challenge because they exhibit a much higher intra-familial sequence similarity than other gene families. The majority (eight out of nine) of Y ampliconic gene families are located in palindromes—structures composed of highly similar inverted repeats (arms) around a relatively short unique sequence (spacer). The arms within a palindrome are 99.9% identical to each other, which results in a high sequence identity among paralogous genes located on the arms [1]. The ninth family, *TSPY*, is present as an array of tandem repeats outside of palindromes [1], however its genes still share sequence identity of >99%. It has been hypothesized that the Y chromosome has acquired its ampliconic structure as a way of facilitating gene conversion [6], which can overcome the decay due to a lack of inter-chromosomal recombination [7,8].

Why these ampliconic gene families are preserved on the Y chromosome remains an open question. It has been suggested that this is due to sexual antagonism eventually leading to increased male reproductive fitness [6,7,9]. Sexual antagonism is expected to lead to the accumulation of genes and mutations benefiting males on the Y chromosome [10]. Consistent with the sexual antagonism hypothesis, all ampliconic genes on the Y are expressed exclusively or predominantly in testis. However, it is also possible that these genes have recently evolved under relaxed functional constraints. The ability to analyze the expression levels of Y ampliconic genes at a large scale can help in exploring their potential functional constraints via comparing their testis expression level to that of their non-Y homologs (when available). For instance, if a Y ampliconic gene family undergoes neo-functionalization, then its resulting expression level is expected to be independent of and potentially higher than that for its non-Y homologs (which we assume retained the ancestral function).

In support of some functional constraints is the observation that the loss or partial deletion of Y ampliconic gene copies is linked to infertility in humans. For example, *TSPY* copy number was linked to both infertility [11] and sperm count [11–13]. The long arm of the human Y chromosome includes three azoospermia factor regions (AZFa, AZFb, and AZFc), which cover most of the ampliconic genes families and are active during different phases of spermatogenesis [14]. Complete or partial deletion of these regions is linked to azoospermia and arrest of spermatogenesis [2,12,14–16]. Presumably, copy number decrease linked with infertility is accompanied by a reduction in gene expression of the affected Y ampliconic gene families, however this is yet to be demonstrated.

Recent studies indicated high variation in Y ampliconic gene copy number in healthy men [3–5]. Skov and colleagues [4] studied Y ampliconic gene copy number variation in 62 men of Danish descent and identified multiple copy number changes across all nine gene families among unrelated individuals, as well as copy number differences for the *TSPY* and *VCY* gene families between a father and a son. Ye and colleagues [3] assessed Y ampliconic gene copy number variation in 100 individuals from around the world. They observed that the size of gene family is correlated with its variation in copy number: larger families, such as *TSPY* and *RBMY*, have higher levels of variation, however the variation appears to be independent of the Y haplogroup. Two men rarely had the same Y ampliconic gene copy number profile and, when they did, this was likely a result of homoplasy. Lucotte and colleagues [5] used the data from the Simons Genome Diversity Project [17] and observed substantial variation in copy number in six out of nine human Y ampliconic gene families [5]. Teitz and colleagues [18] assessed copy number of full-length Y chromosome amplicons located in the AZFc region in men sequenced by the 1000 Genomes Project [18]. Their results suggest that selection has preserved the ancestral ampliconic gene copy number on the Y chromosome in diverse human lineages [18].

These multiple studies of copy number notwithstanding, there has been little investigation of gene expression of Y ampliconic genes. A recent study investigating the expression of Y ampliconic genes during male meiosis found that gene families with high variation in copy number also have high expression levels at different stages of sperm development [5]. Other than the results of this single study, there is a big gap in our understanding of variation in expression of Y ampliconic genes among humans, even though gene expression could be a better predictor of genes’ functions than copy number. Additionally, previous studies have reported that aging affects gene expression [19,20].

Even less is known about how variation in copy number of Y ampliconic genes affects their gene expression. Most parsimoniously, a gain of a complete gene copy should lead to an increase in gene expression levels, unless the extra copy obtains a new function through neo-functionalization, has decreased functional demands due to sub-functionalization or is lost due to pseudogenization. Indeed, this parsimonious hypothesis was supported by the data from the 1000 Genomes Project, where most genes overlapping multiallelic copy number variations (CNVs) display a positive correlation between copy number and gene expression [21]. However, studies across different model organisms have reported that differences in copy number result in increased, decreased or unchanged expression levels among individuals in a population [22]. This more complex relationship can be caused by several scenarios during duplication. For instance, a tandem duplication event may not include regulatory elements, may physically disrupt topologically associated domains (TADs), which prevents the interaction of the gene with its enhancer in 3D space [23,24], or may result in a new copy acting as a negative feedback loop to reduce transcription [22]. Moreover, a non-tandem duplication may occur to a site that is not transcriptionally active [22]. Which of these parsimonious or more complex scenarios occurs on the human Y chromosome ampliconic genes has not been explored.

In this study, we explored the above questions by analyzing the largest data set available to-date consisting of expression data from testis, along with matched whole-genome sequencing data, from 170 men, as generated by the Genotype Tissue Expression (GTEx) consortium [25]. Simultaneously, we developed a novel computational tool AmpliCoNE to estimate the copy number of an ampliconic gene family from sequencing data. Such estimation is complicated by the presence of multiple highly-similar gene copies in the reference, which makes conventional tools inapplicable [26]. Custom strategies have been developed and shown to be effective [4,5,21,27–29], but we did not identify any existing software that could be run directly on Y chromosome ampliconic gene families.

Using AmpliCoNE, we explored whether variation in Y ampliconic gene expression levels could be explained by variation in gene copy number, Y haplogroup, and individual’s age. We correlated the estimated with AmpliCoNE copy numbers of Y ampliconic gene families to their expression levels in testis, and studied how this correlation is affected by Y haplogroups. Additionally, we investigated how testis-specific expression of Y ampliconic genes diverged from their non-Y homologs during evolution.

## Results

### AmpliCoNE: Ampliconic copy number estimator

AmpliCoNE is composed of two programs. The first (AmpliCoNE-build) is executed only once to process the reference genome. It takes the location of all the gene copies in the reference genome, grouped by family, determines which positions in the genes are *informative* (i.e. where read depth is an effective predictor of copy number) and which positions in the reference can be used as a *control* (where copy number variation is infrequent and the read depth has limited noise). The second step (AmpliCoNE-count) is then executed separately for every sample. It parses read alignments and measures the GC-corrected read depth at the informative positions. It then accumulates this information at a family-level and reports the copy number for each gene family, using the read depth at control positions as a baseline. We provide further details in the Methods.

To evaluate AmpliCoNE’s accuracy, we ran it on simulated data and whole-genome short-read data from the Genome in a Bottle (GIAB) consortium [30]. Using the hg38 human genome reference, we simulated three datasets with varying copy numbers of *RBMY*, *TSPY*, and *VCY* gene families and kept the copy numbers for the remaining six gene families constant (i.e. with the copy number found in the reference). AmpliCoNE estimated ampliconic copy numbers correctly 100% of the time in the simulated datasets (S1 Table). We then compared gene family copy numbers between different GIAB experimental runs (technical replicates) for the same human sample (S2 Table), as well as between a father and a son (which can be treated as biological replicates because copy number differences between generations are expected to be rare [4]). AmpliCoNE consistently predicted copy numbers with a difference of less than 0.5 copies per family. We tested AmpliCoNE at different depths of coverage and showed that it can predict similar copy numbers (estimates with difference of less than 0.5) even for datasets with the Y chromosome sequencing depth as low as 6x (S3 Table). AmpliCoNE’s runtime is dependent on the number of reads it needs to process. For instance, it took AmpliCoNE 11 minutes to process the GTEx Y-chromosome-specific BAM file (~500 MB in size).

To measure the concordance between AmpliCoNE’s copy number estimates and complementary non-sequencing assays, we used droplet digital PCR (ddPCR). Both AmpliCoNE and ddPCR were applied to estimate Y ampliconic gene copy numbers for four males sequenced by the GIAB consortium (Tables 1 and S4) [30]. The ddPCR estimates were identical to AmpliCoNE estimates for five out of nine gene families (*BPY2*, *DAZ*, *HSFY*, *PRY*, and *XKRY)* in all four samples. The *CDY* and *RBMY* family copy numbers differed between the two methods in only one and two individuals, respectively. The *VCY* and *TSPY* family copy number estimates differed in three and four individuals, respectively. Compared with ddPCR, AmpliCoNE consistently underestimated the copy number for the *VCY* gene family. Previous studies have indicated presence of X-to-Y gene conversion between *VCX* and *VCY* *[31,32]*. We investigated this case in more detail and discovered that genes from the *VCY* family harbor only a very short (220-bp) sequence distinguishing them from their *VCX* paralogs. This sequence has a low sequencing depth even after GC correction, which results in the underestimation of the *VCY* copy number by AmpliCoNE. In the case of *TSPY*, it is known to have many highly-similar pseudogene copies which may themselves vary in copy number, which can potentially confound both AmpliCoNE and ddPCR estimates. These caveats notwithstanding, AmpliCoNE’s biases in estimating copy numbers for *TSPY* and *VCY* are consistent across samples and thus should not affect our results in a systematic way.

**Table 1:** Experimental validation of AmpliCoNE with droplet digital PCR (ddPCR). AmpliCoNE-based and ddPCR-based Y ampliconic gene copy number estimates of the Ashkenazim and Chinese father-son pairs for the samples analyzed by the GIAB consortium. Differences between AmpliCoNE and ddPCR estimates of >0.5 copy are shown in bold. Differences of >1 copy between a father and a son are underlined.

Gene Family	GM24385 HG002 (A. Son)		GM24149 HG003 (A. Father)		GM24631 HG005 (C. Son)		GM24694 HG006 (C. Father)	
	AmpliCoNE	ddPCR	AmpliCoNE	ddPCR	AmpliCoNE	ddPCR	AmpliCoNE	ddPCR
***BPY2***	2.93	2.82	2.92	3	1.85	1.9	1.91	1.94
***CDY***	**4.34**	**3.47**	4.09	3.68	3.08	2.85	3.10	2.91
***DAZ***	3.86	3.8	3.86	3.99	1.93	2.01	1.85	1.86
***HSFY***	2.01	1.85	2.20	1.88	2.20	1.95	2.15	1.96
***PRY***	2.02	1.92	1.84	2.11	1.82	2.11	2.04	2.02
***RBMY***	5.84	6.72	6.01	6.49	**6.43**	**7.88**	**6.98**	**7.95**
***TSPY***	**39.98**	**42.36**	**39.67**	**44.27**	**20.20**	**21.95**	**19.31**	**21.96**
***VCY***	1.46	2.06	1.59	2.24	1.42	2.09	1.30	2.08
***XKRY***	1.97	1.77	2.16	1.93	1.93	1.91	1.80	1.91

### Y ampliconic gene copy number estimates

Using AmpliCoNE, we estimated copy numbers of Y chromosome ampliconic genes in 170 presumably healthy men whose genomes were sequenced in their entirety as part of the GTEx project [25]. These individuals (S5 Table) were selected because they had matched testis expression data. The individuals belonged to ten major haplogroups: B, E, G, I, J, L, O, Q, R, and T (Table 2). The majority of the samples in the dataset had European or African Y haplogroups, with a few Asian haplogroups present. We also used AmpliCoNE to estimate the copy number of X-degenerate genes, which are expected to be 1 in healthy samples. Three samples had copy number estimates close to zero for two or more ampliconic gene families, or had less than one copy for several X-degenerate genes, which could suggest an individual with a disease or could result from a technical artifact, and thus were removed from the downstream analysis. As a result, we retained 167 samples.

**Table 2:** Y haplogroups and geographic origin for 170 samples used in the study. The numbers after outlier removal (for the remaining 149 samples) are shown in the parentheses.

Major Y haplogroup	Sample size	Sub—haplogroup	Sample size	Major geographic location
R	95(85)	R1a	9(8)	Europe
		R1b	86(77)	
I	29(24)	I1a	19(15)	Europe
		I2a	10(9)	
E	25(22)	E1b	24(22)	Africa
		E2b	1(0)	
J	12(11)	J1a	5(5)	Western Asia
		J2a	5(4)	
		J2b	2(2)	
G	3(2)	G2a	2(1)	Africa
		G2b	1(1)	
T	2(2)	T1a	2(2)	Western Asia
O	2(1)	O1b	1(1)	Eastern and Southeastern Asia
		O2a	1(0)	
Q	1(1)	Q1a	1(1)	Central Asia
B	1(1)	B	1(1)	Africa
Total	170(149)		170(149)	

**Fig 1:**
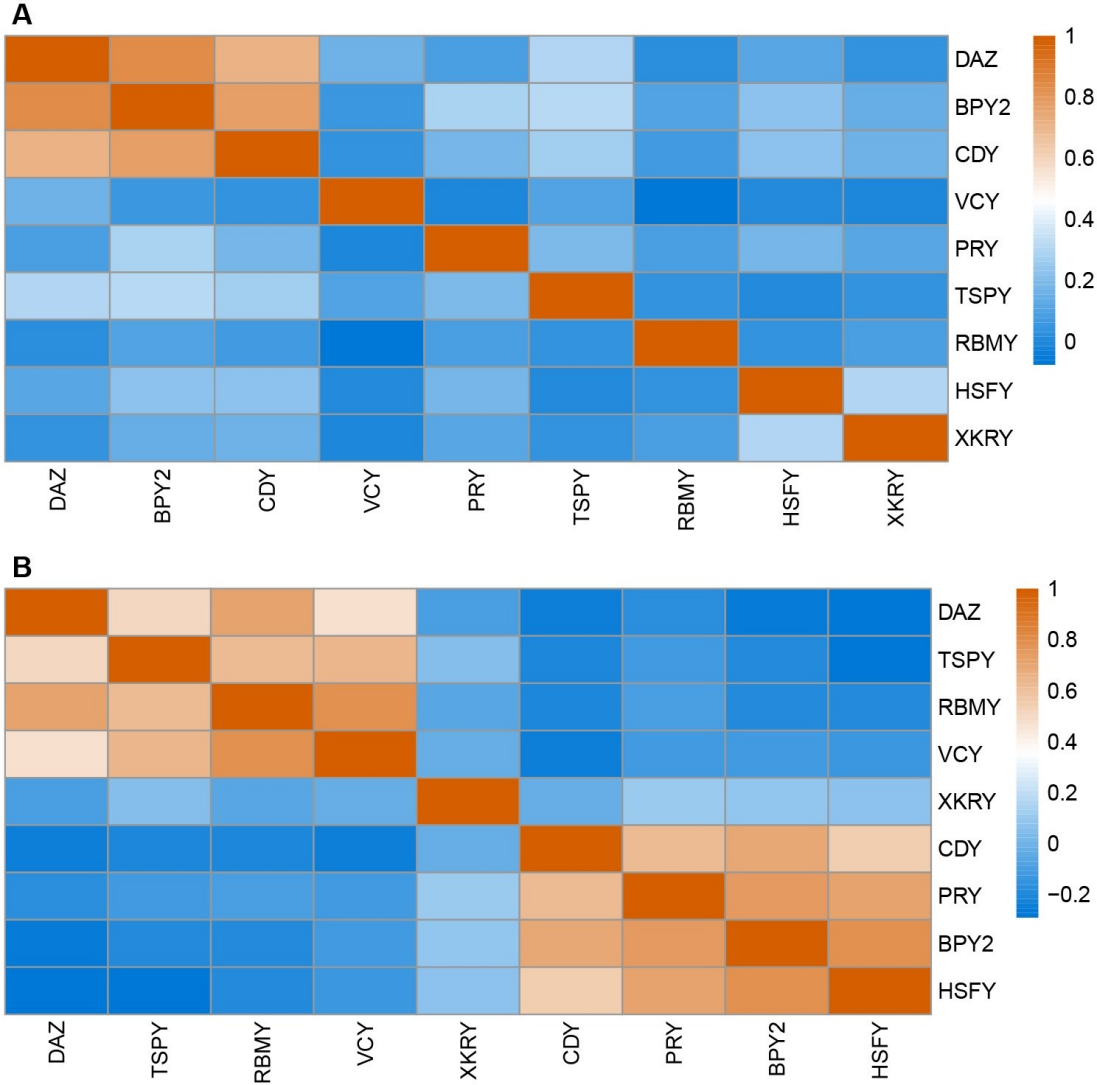
Correlation in copy number and expression levels across Y ampliconic gene families. The gene families are clustered based on correlation coefficients. **(A)** Correlation in copy numbers among 167 individuals. **(B)** Correlation in gene expression levels among 149 individuals.

Gene families with higher median copy number had higher variation when compared to gene families with lower median copy number (*R*^2^ = 0.91; S1 Fig). *RBMY* and *TSPY* were the largest gene families and displayed the highest variation in copy number (5–14 and 20–64 copies for *RBMY* and *TSPY*, respectively). *HSFY*, *PRY*, *VCY*, and *XKRY* were the smallest gene families, which on average had two copies per individual, and displayed low variation in copy number. We observed a positive correlation in copy number among *BPY2*, *CDY*, and *DAZ* gene families, which could be explained by their co-localization on palindrome P1; duplication or deletion involving P1 can affect the copy numbers of all three gene families (Fig 1A).

### Y ampliconic gene families with low copy number in humans are frequently deleted in non-human great apes

We expected to observe a higher probability for gene families with lower median copy number to be completely deleted due to random rearrangements. Therefore, we aimed to test whether the gene families with lower copy number in human had a higher chance of being deleted in non-human great ape species. It is known from previous studies that the *VCY* gene family is missing in gorilla, and orangutan, whereas the *HSFY*, *PRY*, and *XKRY* families are missing in chimpanzee [33]. Consistent with our hypothesis, the *HSFY*, *PRY*, *VCY*, and *XKRY* gene families had low copy numbers in humans (S1 Fig; S6 Table).

### Y ampliconic gene expression

To explore the relationship between ampliconic gene copy number and their expression levels, we analyzed testis expression data from the same 167 humans whose Y ampliconic gene copy number was estimated with AmpliCoNE. After removing outliers (see Materials and Methods), we retained 149 samples and obtained expression levels for each gene family*—*the sum of expression of all the gene copies within each family*—*in each of them. We found that, similar to our observation for copy numbers (S1 Fig), families with higher gene expression levels had higher variation in gene expression (*R*^2^ = 0.99; S2 Fig). The *TSPY* family had the highest gene expression level and the highest variation in expression across individuals, and *XKRY—*the lowest (S6 Table; S2 Fig). The *XKRY* gene family could be considered to be not expressed (as its expression levels are zero) in 58 individuals or expressed at very low levels (with DESeq2 normalized read count < 10) in the remaining 91 individuals. *DAZ*, *HSFY*, and *RBMY* gene families had similar median expression levels and variance among themselves (S6 Table; S2 Fig). Within our dataset, we found two sets of ampliconic gene families whose expression levels were positively correlated with each other (Fig 1B). The first set included *BPY2*, *CDY*, *HSFY*, and *PRY*, and the second set*—DAZ*, *TSPY*, *RBMY*, and *VCY* (Fig 1B). The expression of these sets of gene families could be co-regulated or might have cell-type specificity.

### More copious gene families have higher gene expression levels

**Fig 2:**
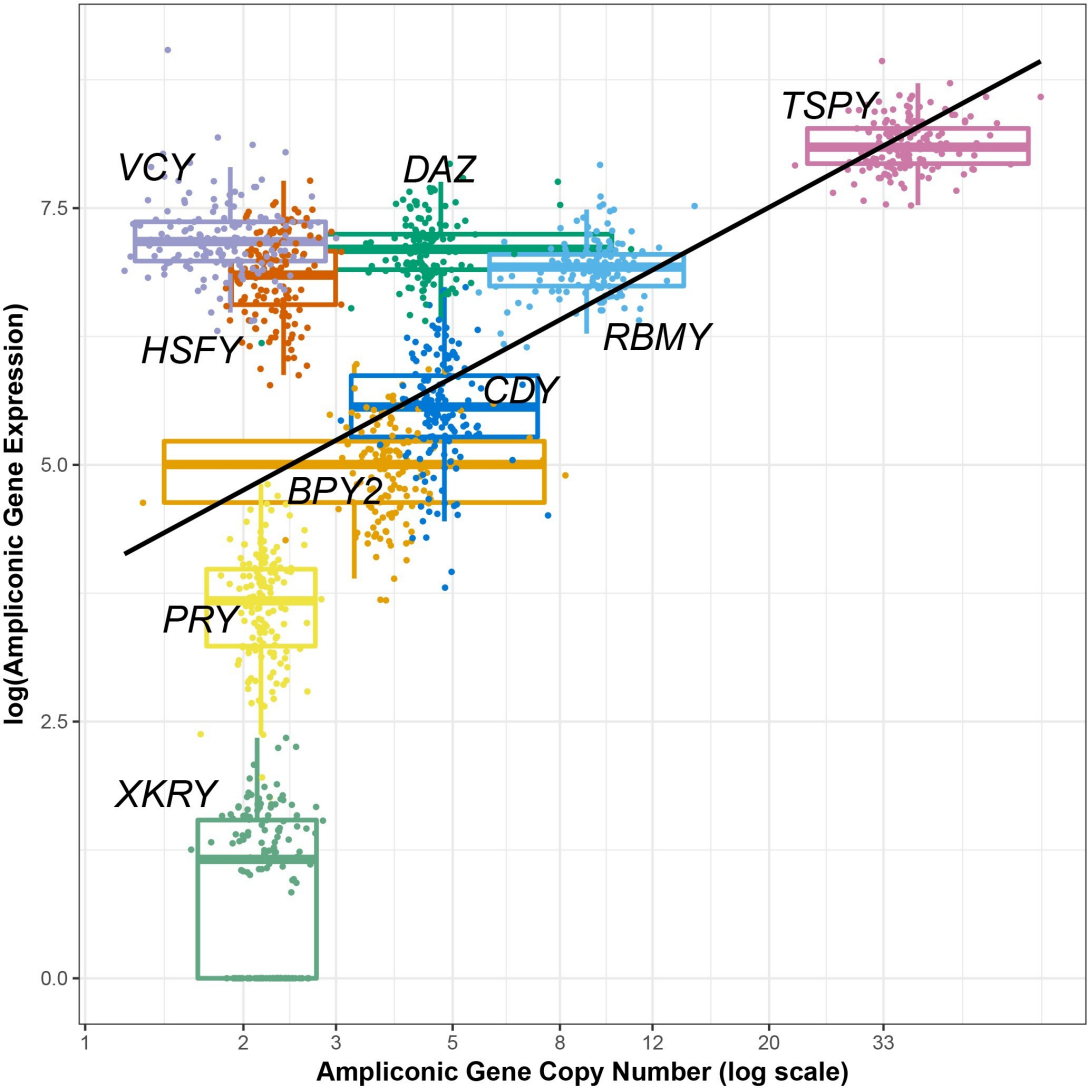
Relationship between copy number and expression levels for nine Y ampliconic gene families. The copy number (X-axis) and gene expression values (Y-axis) values for 149 individuals are presented on a natural log scale. The dots are values for different men, and boxplots are the distribution of values for individual gene families. Both the dots and boxplots are color-coded by their respective gene families. The black line represents the linear function (copy number ~ expression) fitted to the points on the plot. The coefficient of determination (*R*^2^) for the linear model is 0.25.

When we investigated the relationship between expression levels and copy number among all 149 individuals across nine ampliconic gene families, we found that more copious gene families tended to have higher expression levels in comparison to the less copious gene families (Fig 2). Indeed, the expression levels were positively correlated with estimates of copy numbers (Spearman's rank correlation rho = 0.43; *P*-value < 2.2x10^-16^). The *DAZ*, *HSFY*, and *VCY* gene families appeared to be outliers in this analysis, as they had gene expression levels similar to the *RBMY* gene family even though their median copy number estimates were approximately half or less than half of *RBMY* gene family. The *DAZ* gene family had similar gene copy number yet higher expression levels when compared to the *CDY* gene family. The *XKRY* family consistently had very low expression levels, even though its median copy number per individual was two.

### Within a family, copy number and gene expression are not correlated

Next, we tested whether copy number, as measured for each individual, is positively correlated with gene expression levels, again measured for each individual, within the same gene family. There was no significant correlation in any of the nine gene families studied (all *P*-values were above the Bonferroni-corrected *P*-value cutoff of 0.05/9 = 0.006; S3 Fig; S7 Table). To control for genetic variation on the Y, we next compared copy number estimates to gene expression levels for individuals with the same Y subhaplogroup. We focused on the European R1b and I1a, and the African E1b subhaplogroups because they had more than 10 individuals in our dataset (77, 15 and 22, respectively; Table 2). We still found no significant correlations between copy number and expression levels in any of the nine gene families for individuals from either of these three subhaplogroups (all *P*-values were above the Bonferroni-corrected *P*-value cutoff of 0.05/9 = 0.006; S4–S6 Figs; S7 Table).

### Y haplogroups and ampliconic gene families

**Fig 3:**
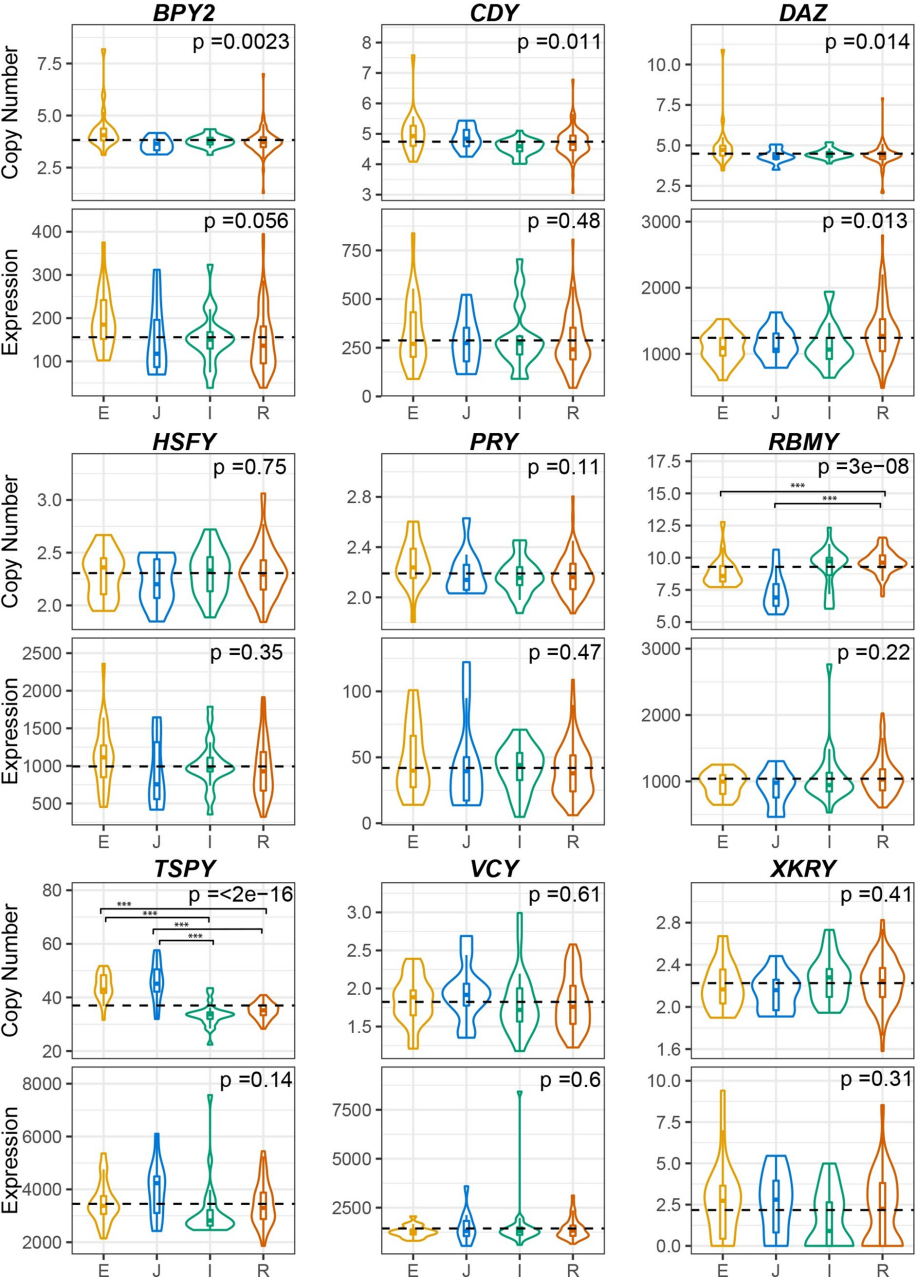
The distribution of ampliconic gene copy numbers and expression levels across Y haplogroups. For each plot the x-axis shows Y haplogroups: E—African (N = 22, yellow), I—European (N = 24, green), J—Western Asian (N = 11, blue), and R—European (N = 85,red), and the y-axis shows copy number estimates or gene expression levels. The black dashed line represents the overall mean copy number or expression values for all the samples on each plot. The permutation-based significance of pairwise haplogroup comparisons is shown with stars (*** < 0.001, ** < 0.01, * < 0.05 *P*-value). The one-way ANOVA test *P*-values are printed at the top of each plot. Bonferroni-corrected cutoff for nine tests (0.05/9 ≈ 0.006) is used to identify significance of ANOVA.

We further asked whether the major Y haplogroup could at least in part explain the variation we observed in copy number and in gene expression levels of Y chromosome ampliconic genes. We focused our analysis on major haplogroups R (European), I (European), E (African), and J (Western Asian) because they were represented by at least 10 samples in our dataset (Table 2). Using one-way ANOVA, we found that the copy numbers of *BPY2* (*P* = 2.34x10^-3^), *RBMY* (*P* = 2.97x10^-8^), and *TSPY* (*P* = 1.07x10^-22^) gene families had significant differences among the four major Y haplogroups analyzed (Bonferroni-corrected *P*-value cutoff of 0.05/9 = 0.006; Table 3). The remaining six gene families did not display significant differences among Y haplogroups (Table 3). When we compared the mean copy number differences between haplogroups in a pairwise fashion using a permutation test (1 million permutations; 9 gene families are tested for 6 cases—R vs E; R vs I; R vs J; I vs E, I vs J, E vs J*—*thus we performed 9 x 6 = 54 tests; Bonferroni-corrected *P*-value cutoff of 0.05/54 = 0.00093), *TSPY* differed significantly in copy numbers (Fig 3) between major European (R and I) vs. African (E) or vs. Western Asian (J) haplogroups (*P* = 0 for R vs. E; *P* = 0 for I vs. E; *P* = 0 for R vs. J; *P* = 0.3x10^-5^ for I vs. J; S8 Table). *RBMY* copy numbers differed significantly between European (R) vs. African (E) or Western Asian (J) haplogroups (*P* = 6.94x10^-4^ for R vs. E; *P* = 0 for R vs J; S8 Table). No significant differences between the two major European haplogroups (R and I) were observed (S8 Table).

**Table 3:** Analysis of variance (ANOVA) of the Y ampliconic gene copy number and expression levels across haplogroups. Conventional one-way ANOVA was performed on copy number estimates and gene expression levels to determine which ampliconic gene families vary in their copy number or gene expression significantly among major haplogroups. F-statistic is computed for one-way ANOVA. *P*-values that pass a Bonferroni-corrected cutoff for nine tests (0.05/9 ≈ 0.006) are highlighted in bold.

Gene family	F-statistic for copy number	P-value for copy number	F-statistic for gene expression	P-value for gene expression
***BPY*2**	5.06	**2.34x10**^**-3**^	2.58	5.61x10^2^
***CDY***	3.89	1.05x10^-2^	0.824	0.482
***DAZ***	3.68	1.37x10^-2^	3.70	1.33x10^2^
***HSFY***	0.411	0.746	1.10	0.349
***PRY***	2.06	0.109	0.848	0.469
***RBMY***	14.48	**2.97x10**^**-8**^	1.51	0.215
***TSPY***	52.48	**1.07x10**^**-22**^	1.86	0.140
***VCY***	0.614	0.607	0.618	0.604
***XKRY***	0.961	0.413	1.22	0.306

In contrast, we found that gene expression levels of all nine Y ampliconic gene families were not significantly different among major Y haplogroups (all *P*-values were above the *P*-value cutoff of 0.05/9≈0.006; one-way ANOVA; Table 3). We observed a trend suggesting differences in expression values among haplogroups for the *BPY2* and *DAZ* gene families, but these differences were small in scale. Nevertheless, out of the nine gene families, *BPY2* (*P* = 0.056) and *DAZ* (*P* = 0.01) had low *P*-values for the ANOVA analysis (Table 3, Fig 3) and for the permutation test comparing mean expression levels between haplogroups (*P* = 7.09x10^-3^ for E vs. R for *BPY2*; *P* = 1.36x10^-2^ for E vs. R for *DAZ*; *P* cutoff of 0.05/54 = 0.00093; S9 Table). When we compared the trend in copy number and gene expression differentiation among haplogroups, we observed that in the *TSPY* gene family both copy number and gene expression levels were lower for the European haplogroups (I, R) than for the African (E) or Western Asian (J) haplogroups (Fig 3). This trend was statistically significant for copy number, but not significant for gene expression. Analyzing a larger sample size might lead to finding this trend to be significant also for gene expression.

### The role of age in ampliconic gene expression

To examine the potential role of aging in determining Y ampliconic gene expression, we compared the ages of individuals at the time of sample collection to the ampliconic gene expression levels and found no statistically significant relationship (nine gene families were tested for correlation which results in Bonferroni correction *P*-value cutoff of 0.05/9 = 0.006; S7 Fig; S10 Table). Next, to perform a similar analysis for individuals with the same subhaplogroup, we limited our analysis to individuals with the European R1b and I1a, and African E1b subhaplogroups (77, 15, and 22 individuals, respectively). For the R1b and I1a subhaplogroups we found no significant relationship between age and expression levels for any of the nine Y ampliconic gene families studied (S8 and S9 Figs; S10 Table). However, for the African E1b subhaplogroup, *HSFY* (Spearman correlation = 0.57; *P* = 0.0061) and *PRY* (Spearman correlation = 0.61; *P* = 0.0028) gene families had a positive correlation between expression levels and age, which was significant after Bonferroni correction (S10 Fig; S10 Table). A larger dataset of African samples should be studied to validate this relationship.

### Ampliconic gene dosage regulation

The presence of homologs outside of the Y for two groups of Y ampliconic gene families allows us to study evolution of their gene expression levels [34]. In particular, the *CDY* and *DAZ* genes were copied to the Y chromosome from autosomes [34]; the *HSFY*, *RBMY*, *TSPY*, *VCY*, and *XKRY* gene families have homologs on the X and were likely present on the ancestral autosomes giving rise to the two sex chromosomes [34]. In the analyses below, we assume that the testis-specific expression of Y ampliconic genes was acquired prior to their amplification on the Y [9] and that their autosomal or X-chromosomal homologs have maintained ancestral expression levels, i.e. they possess expression levels of Y ampliconic genes prior to their Y linkage [35]. The latter assumption is based on the overall slower rates of evolution of X-chromosomal and autosomal genes as compared to their Y-chromosomal homologs.

**Fig 4:**
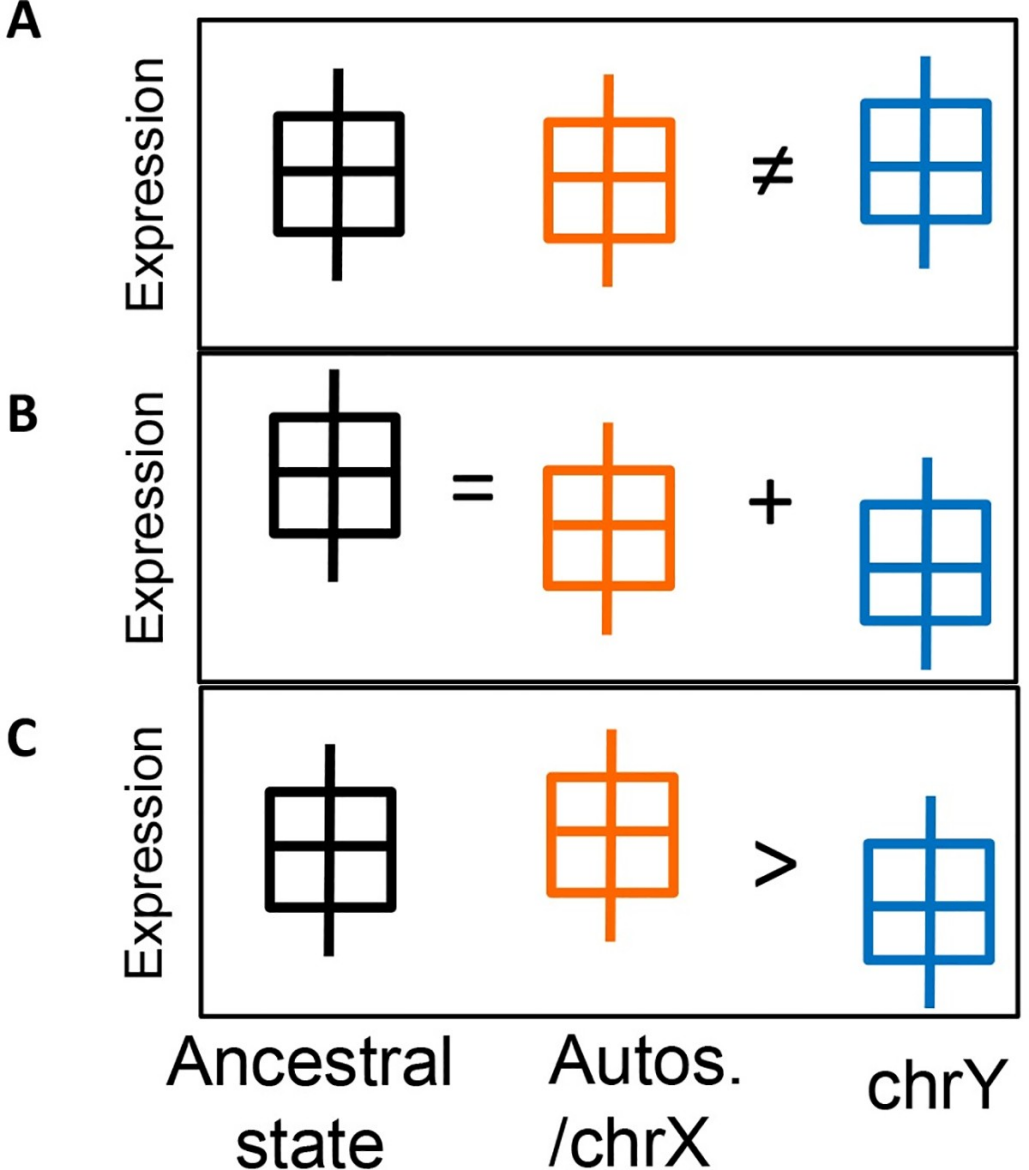
Possible differences in expression between Y ampliconic genes and their non-Y homologs. **(A)** Neo-functionalization: Ampliconic gene family, after moving to Y or divergence from the X, obtained a new beneficial function. The gene family might be under positive selection and its expression might be independent from its non-Y homolog. **(B)** Sub-functionalization: Ampliconic gene family, after moving to the Y or divergence from the X, acquired a (testis-specific) sub-function. Because sub-functionalization is division of labour, the sum of ampliconic gene expression and non-Y homolog expression should be equal to the ancestral expression. The average expression of ampliconic gene family could be lower or higher than that of their homologs and depends on the subfunction. **(C)** Relaxed selection: Ampliconic gene family, due to its multi-copy nature and presence of gene conversion, evolves at a faster rate than its non-Y homolog and is not under selection.

We envision three possible scenarios for gene expression evolution of Y ampliconic gene families that have non-Y homologs (Fig 4). First, because of sexual antagonism, a gene on the Y could obtain beneficial mutations and diverge in function from its non-Y homolog to acquire new functions in testis (i.e. neo-functionalization). The expression of such a gene family would be independent from, and potentially higher than that for, its non-Y homologs (scenario A). Second, a gene family on the Y could retain function of the non-Y homolog, but acquire testis-specific expression (i.e. sub-functionalization). In this case, either the non-Y copy represents the ancestral expression levels and the Y copies are expected to maintain low expression levels, or the sum of expression from the Y and non-Y copies is regulated to be at levels similar to those of the non-Y copy in the ancestor (scenario B). In this case, the expression of both Y and non-Y homologs might be down-regulated. Third, genes on the Y might be under relaxed selective constraints and thus have low expression levels (scenario C) [36]. Below we test these three scenarios by comparing expression levels of both Y and non-Y ampliconic gene homologs in testis tissue.

In addition to the analysis of such overall differences in the expression level (Fig 4), we can also examine the relationship between the Y ampliconic genes’ and their non-Y homologs’ gene expression across individuals, which should further assist in determining a particular evolutionary scenario (S11 Fig). If the expression levels of Y ampliconic genes are higher than those of their non-Y homologs, and across individuals the expression levels of these two groups of genes are positively correlated, then this pattern is consistent with neo-functionalization of the Y ampliconic genes. This is because higher expression levels of ampliconic genes than those at the ancestral state suggest independent expression of Y ampliconic genes from their non-Y homologs, and a positive correlation between Y ampliconic genes and their non-Y homologs suggests co-regulation, e.g. they might share similar transcription factors [37]. A combination of these two patterns suggests an acquisition of a new function (scenario A) (S11A Fig). If the expression levels of Y ampliconic genes are higher than those of their non-Y homologs, and across individuals the expression levels of these two groups of genes are negatively correlated, then the data are compatible with neo- or sub-functionalization (scenario A or B). Indeed, the observed negative correlation could be explained by neo-functionalization, where ampliconic genes acquired a new function and inhibit the expression of the non-Y homologs. Alternatively, the negative correlation could be explained by sub-functionalization, where ampliconic genes acquired new transcription factors which limit their expression to a few cell types, and the negative correlation is due to the differences in the abundance of cell types in which ampliconic genes are expressed (S11B Fig). If the expression levels of Y ampliconic genes are lower than those of their non-Y homologs, and across individuals the expression levels of these two groups of genes are positively correlated, then this pattern is consistent with any of the three scenarios A-C. This is because the lower expression levels of Y ampliconic genes could be due to down-regulation of gene expression by the Y chromosome to accommodate the multi-copy state of ampliconic genes [38], evolution of which could still be compatible with any of the three scenarios A-C (S11C Fig). If the expression levels of the Y ampliconic genes are lower than those of their non-Y homologs, and across individuals the expression levels of these two groups of genes are negatively correlated, then the data are compatible with scenario A or B. This is because negative correlation eliminates the scenario of relaxed selection, i.e. scenario C (S11D Fig). Finally, if we observe no correlation in expression levels between Y ampliconic genes and their non-Y homologs, then we can conclude that their expression is independent from each other, which could be a result of neo-functionalization, sub-functionalization or random drift in expression levels under relaxed selection.

**Fig 5:**
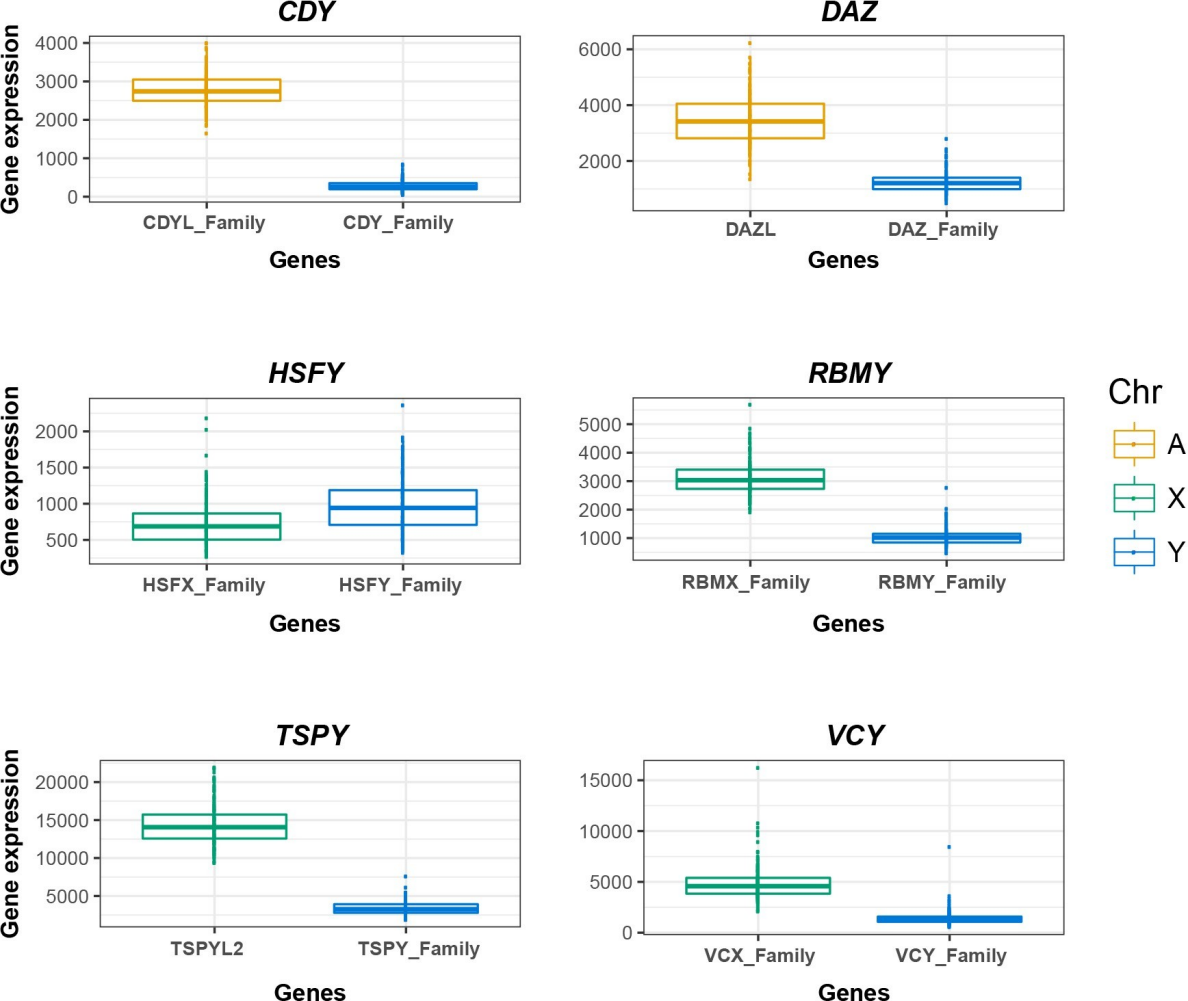
Expression differences between Y ampliconic genes and their non-Y homologs. Each plot compares expression levels of Y ampliconic gene family (the sum of expression of all copies of a gene family, blue) to their homologs on the X chromosome (green) or autosomes (yellow). The gene names are shown on the x-axis and normalized expression levels*—*on the y-axis.

**Fig 6:**
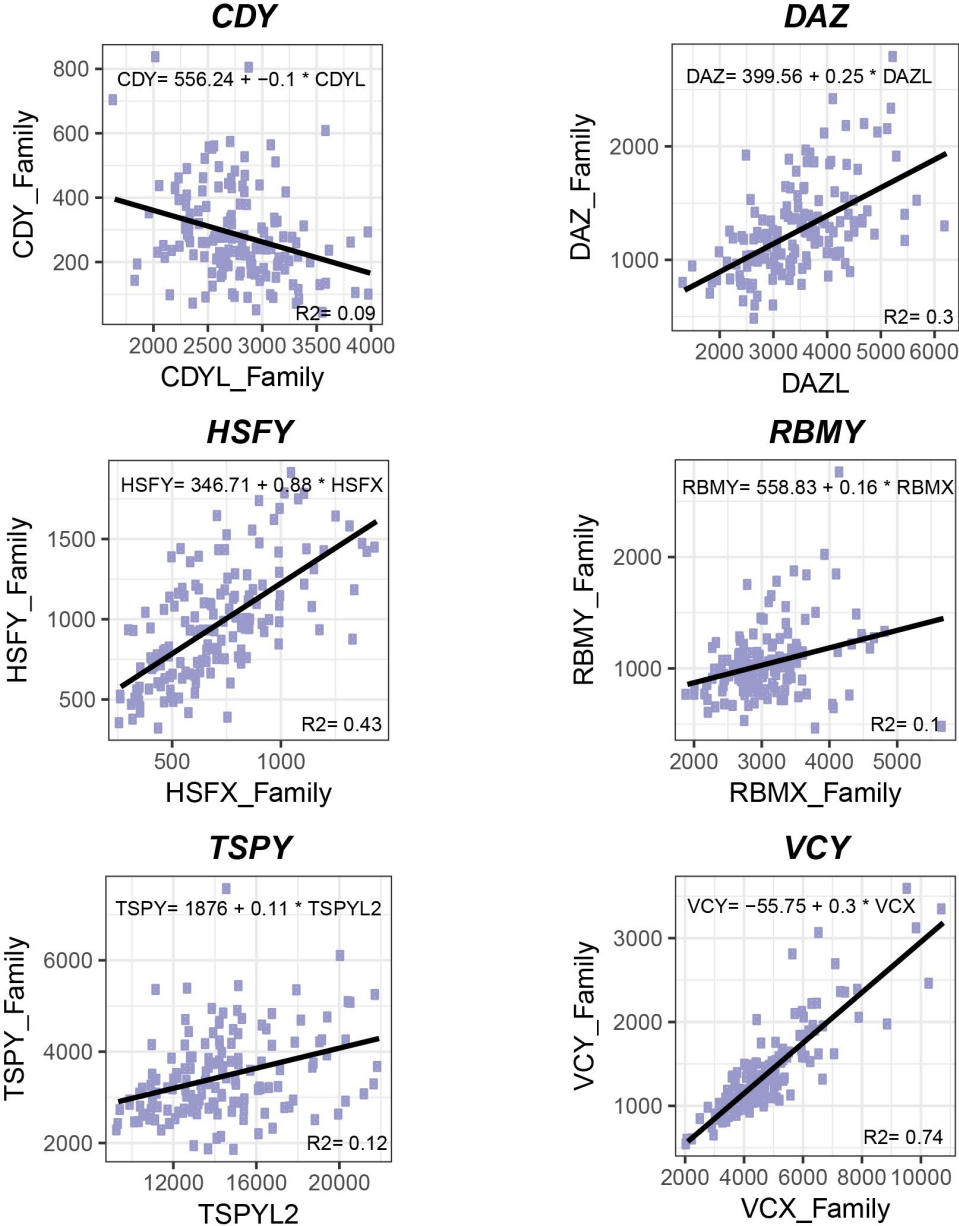
Individual-level relationship between Y ampliconic genes and their non-Y homologs. Each plot compares expression levels of Y ampliconic gene family (the sum of expression of all copies of a gene family) to their non-Y homologs in each individual (N = 149). Each dot represents an individual. The black line represents the linear regression fit to the data and the respective equation is at the top of each plot. The R-squared value is at the right-hand side bottom of each plot.

To test these scenarios, we first compared testis expression levels between Y ampliconic gene families *CDY* and *DAZ*, which were copied to the Y from autosomes, and their autosomal homologs (Fig 5). The *CDY* autosomal homologs *CDYL* and *CDYL2* are ubiquitously expressed; and the *DAZ* autosomal homolog *DAZL* has testis-specific expression [34,39–41]. The expression levels of *CDY* (the sum of expression levels for the whole gene family) were 89% lower than those for their autosomal homologs (the sum of expression of *CDYL* and *CDYL2*), and for *DAZ* they were 63% lower than those for their autosomal homolog *DAZL* (Fig 5). Next, we tested whether the expression levels for Y ampliconic genes and their autosomal homologs are regulated at the level of each individual. For each gene family, we examined a potential correlation in gene expression levels between the Y ampliconic genes and their non-Y homologs. We observed a significant negative correlation between *CDY* and *CDYL*+*CDYL2* expression levels (Spearman correlation = -0.31; P = 2x10^-4^), which indicates that, across individuals, whenever the *CDY* expression levels increase, the *CDYL*+*CDYL2* expression levels decrease (Fig 6). In case of *DAZ*, a positive correlation in expression levels (Spearman correlation = 0.57; P = 0) was observed between *DAZ* and its autosomal homolog *DAZL* (Fig 6). Lower expression of *CDY* and *DAZ* than their non-Y homologs could be a result of down-regulation of gene expression by Y chromosome to maintain the multi-copy state, however the negative correlation in *CDY* vs. *CDYL*+*CDYL2* expression levels indicates the presence of either neo- or sub-functionalization. *DAZ* could have undergone any of the three scenarios, which are difficult to differentiate based on the available data.

We next examined how testis-specific gene expression of the *HSFY*, *RBMY*, *TSPY*, *VCY*, and *XKRY* gene families diverged from that of their X homologs. Most of the X homologs of ampliconic genes (except for *VCY* and *XKRY*) are expressed in multiple tissues along with testis. The *XKRX* gene, an X homolog of the *XKRY* gene family, is not expressed in testis and we omitted this gene family from our analysis (S11 Table). Three Y gene families studied (*RBMY*, *TSPY*, and *VCY*) on average had lower expression levels in comparison to their X homologs (66%, 75%, and 71% lower, respectively; Fig 5). *HSFY* was the only gene family that on average had higher expression in comparison to their homologs on the X (35% higher than X-homologs). This could imply that *HSFY* might have acquired a new function, which is selected for in testis (scenario A). At the level of the studied individuals, all four studied gene families exhibited positive correlation in gene expression levels between their Y ampliconic and X homolog genes, suggesting a potential co-regulation (Fig 6). This correlation was particularly strong for the *HSFY* and *VCY* gene families (Spearman correlation of 0.69 and 0.84, respectively). The observed higher expression of *HSFY* than of its X homologs, as well as positive correlation in gene expression levels between these two groups of genes, is a strong indicator of neo-functionalization. In the case of *RBMY*, *TSPY*, and *VCY*, it is challenging to differentiate among the three scenarios we propose based on the available data.

## Discussion

Ampliconic genes constitute the majority (80%) of protein-coding genes present on the human Y chromosome and play an important role in spermatogenesis [1]. Yet, very little is known about the significance of Y ampliconic gene copy number variation in determining their expression levels in humans. Here we analyzed both copy number and testis-specific expression of ampliconic gene families in 149 presumably healthy men. Our goal was to understand the relationship between copy number variation and expression levels while accounting for Y chromosome haplogroups.

### Variability in Y ampliconic gene copy number

Our results indicate that smaller Y ampliconic gene families maintain lower variation in copy number and, as the size of gene families increases, variation in copy number also increases, in agreement with previous studies [3–5]. The parsimonious explanation for this observation is that a greater number of gene copies leads to loss or gain of gene copies because of a higher probability of rearrangements via replication slippage and/or non-allelic homologous recombination (NAHR) [42–44]. On the human Y, the larger gene families are either spread across multiple palindromes (e.g., *RBMY*) or are arranged as a tandem array (*TSPY*), and such arrangements can result in multiple scenarios of NAHR, which will lead to gain or loss of gene copies. *BPY2* has two functional copies on palindrome P1 and one copy outside of palindromes, and such an arrangement can also result in NAHR.

We found that the large *TSPY* and *RBMY* gene families have not only a high level of variation in copy number, but also a significantly different number of gene copies among the major Y haplogroups analyzed. An earlier study also found significant differences in copy number for these two gene families among human Y haplogroups across the world and suggested that this observation cannot be explained by selection [3]. However, selection explanation might warrant a further investigation. Indeed, a recent molecular analysis of infertile men indicated a positive correlation between the number of *RBMY* copies and sperm count and motility [45]. Moreover, *RBMY* is a male-specific oncogene [46]. Therefore, it will be of interest to investigate whether variation in *RBMY* copy number across Y haplogroups influences these two disease-related phenotypes and might be subject to natural selection. Similarly, *TSPY* is a candidate proto-oncogene which can regulate its own expression via a positive feedback loop in gonadoblastoma and a variety of somatic cancers [47]. Thus, additional studies should be performed to test whether variation in *TSPY* copy number across haplogroups is associated with differential predisposition to gonadoblastoma.

The smaller Y ampliconic gene families (*HSFY*, *PRY*, *VCY*, *and XKRY*) have lower variation in copy number compared with larger families. These gene families, for which the average family size is only two copies, are each present on an individual palindrome (the two copies are present as inverted repeats on opposite palindrome arms). Recombination between inverted repeats is expected to result in an inversion keeping copy number constant [48]. In addition, the presence of only two copies increases the chances of a complete gene family elimination due to Muller’s ratchet or of rearrangements which involve the whole palindrome. Consistent with this prediction, we find these gene families to be deleted or pseudogenized in several great ape species [33].

Thus, the copy number of ampliconic genes is an important factor in determining the survival of a gene family on the Y chromosome. Too few copies can lead to a complete loss of a gene family (see the preceding paragraph), whereas too many copies can lead to frequent NAHR which can rapidly increase or decrease copy number [49]. Consistent with this expectation, it was suggested that the human Y chromosome evolves under selection to maintain an optimal copy number for its amplicons in diverse human lineages [18]. Most likely both random genetic drift and natural selection contribute to determining the Y chromosome ampliconic gene copy number. Drift leads to smaller-scale changes in copy numbers, whereas selection might act at removing extreme copy numbers because too few copies might lead to infertility and too many copies might lead to genetic instability and thus both are selected against. Variation in Y ampliconic gene copy number in subfertile and infertile males should be investigated in future studies and should shed additional light on the balance between these two evolutionary processes.

Note that in the present study we only examined complete gene copy gains or losses, but insertions and deletions inside a gene can also affect gene expression and functionality, and might be linked to infertility [50]. The effects of such smaller CNVs are more robustly evaluated from long-read data and we leave this exploration to future work.

### Variability in Y ampliconic gene expression

Here we studied the expression levels of the Y ampliconic gene families in testis tissue of presumably healthy individuals. The vast majority of cells in testis are germline cells in the seminiferous ducts, where spermatogenesis takes place. We primarily captured Y chromosome gene expression in spermatogonia prior to meiosis and throughout different spermatogenesis stages after meiosis [51,52]; this is because Y transcription is silenced at other stages of spermatogenesis due to meiotic sex chromosome inactivation [52,53] and postmeiotic sex chromosome repression [51,52]. As a tissue, testis is a mixture of germline cells at different stages of development, Sertoli cells, myoid peritubular cells, and interstitial Leydig cells. Thus, the expression values generated using testis tissue as a source represent cumulative gene expression of germline cells at different stages of spermatogenesis with a mixture of somatic cells. This potential limitation notwithstanding, our results indicate substantial variation in expression levels for Y ampliconic genes in testis among men and suggest that different levels of Y ampliconic genes’ expression are tolerated by presumably healthy individuals.

When we compared copy number of ampliconic genes to their gene expression values, we found that *across gene families* the gene families with higher median copy number had higher expression levels. This is consistent with an observation made by Lucotte and colleagues [5] who reported on the expression of Y ampliconic genes at different stages of spermatogenesis with respect to variation in their copy number. Overall, the Y chromosome has higher copy number of genes for those gene families whose median expression levels are higher in testis, however it is important to note that this relationship might be different at individual cell types in testis and should be studied further.

When we examined the relationship between copy number and expression *within a gene family*, our analysis revealed that expression of Y ampliconic gene families is independent of their copy number. Moreover, no significant differences in Y ampliconic gene expression levels were observed among Y haplogroups, even though we found significant differences among Y haplogroups in copy number for some gene families (*BPY2*, *TSPY*, and *RBMY*). This suggests that testis tissue might have evolved the ability to tolerate different Y ampliconic gene copy numbers, and also variable Y ampliconic gene expression levels.

Approximately 77% of all protein-coding genes in the human genome are expressed in testis [54], and some of these genes could regulate expression of the Y ampliconic genes. Understanding the 3D organization and chromatin structure on the Y is expected to aid in identifying the genomic regions and genes that ampliconic genes interact with and are regulated by in the genome. Future studies analyzing expression data at different stages of spermatogenesis in individuals with different Y ampliconic gene copy numbers will assist in deciphering the role of copy number variation in determining gene expression in more detail. Additionally, our findings should be confirmed by studies of gene expression at the protein level.

A man’s advanced age has significant negative impact on reproduction [55]. Semen parameters such as daily sperm production, total sperm count, and sperm viability are negatively correlated with age [56]. However, within our dataset, we observed mixed results regarding age effects on Y ampliconic gene expression: age did not influence variation in gene expression of these genes in individuals with European Y subhaplogroups I1a and R1b, however *HSFY* and *PRY* expression had a positive correlation with age in individuals with an African subhaplogroup E1b. These findings should be validated with a larger data set to examine the role of Y ampliconic genes in changes in spermatogenesis with age.

### Dosage regulation of Y ampliconic genes

The Y chromosome degradation, which is common across eutherian mammals, has resulted in the loss of the majority of genes originally present on the proto-sex autosomal pair [57]. To balance the loss of genes on the Y in males, the mammalian X chromosome adapted its expression levels by inactivating one of its copies and increasing the expression of the other copy in females [57–59]. We wondered whether a similar process evolved at Y ampliconic genes that have non-Y homologs, namely whether the expression of Y ampliconic genes and their non-Y homologs has been co-regulated. Alternatively, Y ampliconic genes might have evolved new functions, and thus potentially high expression levels, independent of their non-Y homologs. Yet another alternative would be the overall low expression levels because of the relaxation of functional constraints on the Y ampliconic genes. The precise functions of Y ampliconic genes have been under-characterized (S12 Table) due to the repeated nature of the Y chromosome and scarcity of testable orthologs in model organisms. While Y ampliconic genes have testis-specific expression likely as a result of sexual antagonism, the majority of non-Y homologs of Y ampliconic genes have ubiquitous expression.

Recently, a multi-step model for preservation of tandem duplicate genes was presented. According to this model, the expression of gene duplicates is down-regulated immediately after the duplication event, followed by dosage sharing which could lead to functional adaptations such as sub- or neo-functionalization [38]. Knowing that non-Y homologs of Y ampliconic genes are expressed in testis (except for *XKRX*), we compared the expression levels of closely related homologs of ampliconic genes on both autosomes and X chromosome to the sum of expression levels for all the copies of a Y gene family. We demonstrated that, with the exception of the *HSFY* family, Y ampliconic gene families have consistently lower expression levels when compared to their non-Y homologs, thus not elevating the overall expression level of the family. We term this phenomenon *dosage regulation of Y ampliconic genes*. Lower expression of Y ampliconic gene families could be an adaptation of the Y to maintain the multi-copy state of ampliconic gene families. By lowering the expression of the whole gene family, the Y can buffer sudden loss or gain of gene copies. In addition to dosage regulation, the gene family should be expressed at optimal levels to maintain their functionality during spermatogenesis. Lower optimal expression of Y ampliconic gene families compared to their non-Y homologs could be a result of sub-functionalization (e.g., testis specificity in expression), which benefits germline cell development. Alternatively, such low expression could be a result of relaxed selection, and, in agreement with this possibility, Y ampliconic genes show a higher rate of nonsynonymous to synonymous substitution rates compared to single-copy X degenerate genes on the Y [7]. Alternatively, a gene family could be under positive selection or undergoing neo-functionalization even in their low-level expression state. The expression of ampliconic gene families is important for spermatogenesis because of an association between gene deletions and infertility, but relaxed selection can facilitate rapid differentiation of ampliconic gene function.

We found that expression levels of the *CDY* ampliconic genes and those of their autosomal homologs are negatively correlated among individual men. This suggests that the *CDY* gene family might not be expressed at the same time during spermatogenesis as its autosomal homologs or that there is a coordinated down-regulation of *CDY* expression with a rise in *CDYL* and *CDYL2* expression (and vice versa). In humans, the *CDYL* and *CDYL2* autosomal genes produce the ubiquitously expressed long transcripts, but lost the testis-specific short transcript which is now produced by *CDY* [40]. The combined tissue expression patterns of *CDY*, *CDYL*, and *CDYL2* in human recapitulate the expression patterns of *CDYL* and *CDYL2* in mouse or rabbit, which do not have *CDY* on their Y chromosome [40].

In contrast with *CDY*, we found that expression levels of *DAZ*, *HSFY*, and *VCY* gene families are strongly positively correlated with their non-Y homolog expression across individuals, which suggests a co-regulation in gene expression levels of these ampliconic gene families and their homologs (the *RBMY* and *TSPY* families also show positive correlation, however it is not strong). When we examine the linear relationship between ampliconic gene families and their homologs among individual men, the Y ampliconic gene expression increases at a slower pace when compared to the expression of their non-Y homologs, except for *HSFY* where the expression increases at a similar rate for both Y and non-Y homologs (Fig 6).

The *VCY* gene family is the most commonly lost gene family among great apes, however in our dataset the expression of this gene family is higher than for most other gene families on the Y and is higher than is predicted from its copy number (Fig 2). The homologs of *VCY* on the X chromosome (*VCX*, *VCX2*, *VCX3A*, and *VCX3B*) are expressed in testis [60,61]—and we show that at higher levels than the *VCY* family itself. In addition, there is high sequence identity (>95%) between the *VCX* and *VCY* gene families, which could imply that both *VCX* and *VCY* could have been under selection to maintain function of the gene family, however, to balance the expression of the multi-copy *VCX* family, *VCY* might have lowered its expression. The role of both VCX and VCY in ribosome assembly in spermatogenesis has been suggested [62]. The loss of *VCY* in great ape species might have been compensated by functionally similar *VCX* family expression in testis. The expression levels of the *VCX* family across great apes must be studied to understand its role in the loss of *VCY*.

A recent study found multiple distinct clusters of full-length Y ampliconic gene transcripts, likely originating from different copies of the same family [63]. Therefore, the presence of multiple full-length transcripts [63] and low expression levels for Y ampliconic gene families (the present study) suggest that individual gene copies within a family are down-regulated to accommodate the expression of the whole gene family on the Y chromosome and outside of it (on autosomes and on the X). This hypothesis needs to be examined in future studies in which expression levels of individual gene copies will be evaluated with long-read sequencing technology. It will also be important to decipher the isoforms and their expression levels for Y ampliconic genes and their non-Y homologs to understand whether Y ampliconic genes and their homologs express the same isoforms, or whether Y ampliconic genes express their own, unique, testis-specific isoforms.

It is essential to note that, in addition to evolution of expression levels of the whole gene family including its non-Y homologs, the Y ampliconic genes can diverge to acquire additional male-specific functionality because they are present on the Y, which is susceptible to accumulating genetic differences dictated by sexual antagonism. In other words, Y ampliconic genes could have gained secondary functions independent of their functions on the proto-sex chromosomes. This scenario might be exemplified by the case of the *HSFY* family, whose expression levels have increased in comparison to its X-chromosome homologs. This pattern suggests that this gene family underwent neo-functionalization. The exact function of *HSFY* is unknown, but its role in transcription regulation has been suggested because it harbors a DNA-binding domain [64]. In fact, it was shown that *HSFY* and *HSFX* share only this DNA-binding domain but not the rest of their sequences and thus indeed might have diverged in their functions [64]. Moreover, *HSFY* has stage-specific expression during spermatogenesis, suggesting that it acquired a function different than that of heat shock proteins it is homologous to [64]. The loss of *HSFY* was linked to infertility [64–66]. In another study, under-expression of *HSFY* was linked to arrest of maturation of nascent germ cells to motile sperm [67]. According to our study, the expression of *HSFY* gene family was positively correlated with age in the African E1b Y haplogroup, however such a relationship was not found in the R1b haplogroup. Further studies addressing transcription regulation by the *HSFY* family in individuals of varying age across different Y haplogroups are required to understand the *HSFY* functionality in more detail.

We assume that non-Y homologs have retained the ancestral expression state because of the overall fast evolutionary rate on the Y chromosome [68]. However, the X chromosome and autosomes have also been evolving, albeit slower than the Y. Evolutionary changes acquired by non-Y homologs since they diverged from the Y homologs have not been addressed in this study due to the lack of ancestral expression data. To address this, future studies should identify species which have orthologs of human ampliconic genes in a single-copy state on their Y chromosome. In the case of *CDY* and *DAZ* gene families, future studies should identify species in which these genes’ orthologs are present in a single-copy state on their autosomes and absent from the sex chromosomes. Once such species are identified, their testis-specific expression data for these genes could be used as the ancestral expression state.

## Materials and methods

### AmpliCoNE: Ampliconic copy number estimator

To estimate copy number in highly-similar multi-copy gene families, several strategies have been proposed. One can align each read to all possible locations in the reference genome [69], identify sites in the reference genome that uniquely distinguish and tag paralogs of interest [18,21,27,29], use simulated reads for mock genomes with human gene cDNAs at different gene copy counts to obtain a theoretical function of the coverage distribution with respect to copy number [28], or customize the reference to keep a single copy of each gene family [4,5]. While these strategies were effective in their respective papers, we could not find software that could work on human Y ampliconic genes. We therefore combine the ideas from these strategies into AmpliCoNE, a tool for estimating copy number in highly-similar multi-copy gene families. The Results section contains an overview of AmpliCoNE, but we provide more details here.

#### Determining control and informative positions

To calibrate a baseline for read depth at copy number of one, AmpliCoNE uses single copy regions unique to the Y (AmpliCoNE also provides an option to use X-degenerate regions as a control, but we do not describe it in the manuscript). These regions are identified by AmpliCoNE-build as positions in the Y chromosome such that the k-mer starting at the position does not match any other location in the reference. We used a k-mer of size 101, since this is the length of the shortest reads that we use from GTEx, and we allowed up to two edits in matching to other locations. AmpliCoNE-build computes these positions by using the GEM-mappability tool (v1.315) [70] and extracting those locations with a mappability of 1.

AmpliCoNE-build pre-determines which positions of the genome will be used for later measuring read depth. A position within a gene family is said to be *informative* if (1) the location is not within an annotated high-copy repeat region (e.g. a transposable element) or a short tandem repeat, (2) the k-mer starting at that location is specific to its gene family of origin, and (3) the k-mer is non-repetitive within its gene. For (1), AmpliCoNE-build takes repeat annotations as input, such as ones generated by RepeatMasker [71] and Tandem Repeat Finder [72]. This step is necessary since these regions are notoriously hard to align to. For (2) and (3), AmpliCoNE-build uses a strategy similar to the one used by Tietz and colleagues to annotate Y chromosome amplicons [18]. It extracts all the 101-mers from the Y chromosome and maps them back to the reference using Bowtie2 [73]. It allows Bowtie2 to generate up to 15 alignments per k-mer (-k 15) but discards alignments that have more than two mismatches. A k-mer is then determined to be specific to the gene family if all its alignments fall within the gene regions in the family. It is determined to be non-repetitive within its gene if the number of alignments equals to the number of genes in the family. Using only gene-specific locations is crucial for AmpliCoNE's accuracy, since non-specific locations would add biased noise to later read depth estimates.

#### Computing read depth and calling copy number

AmpliCoNE-count takes an alignment file of male reads to the male reference as input. It only retains alignments that are part of a properly mapping read pair and have at least 88 perfect matches in the first 90 bp of the read. This threshold is designed to retain only reliable alignments and is intended to match the criteria used for determining control positions. For each non-repeat-masked position *i* on the Y chromosome, AmpliCoNE-count then computes the number of alignments starting at *i*, which we refer to as the *read depth D_i_*.

It is known that the GC bias affects the depth of reads generated using Illumina technology [74]. Therefore, AmpliCoNE-count adjusts the read depth by using a sliding-window-based GC correction method [75]. Concretely, AmpliCoNE-count first collects the read depths for the control positions and notes the GC percentage of the 501 bp window centered at those positions. The read depths are then binned according to their GC percentage, using 100 bins: for a given bin *b*, we calculate *μ*_*b*_, the mean read depth of the control locations with a GC percentage belonging to *b*. We also let *μ* be the mean read depth over all control positions. The GC-corrected read depth for position *i* is then calculated as *μD_i_/μ_b_*.

For each gene, AmpliCoNE-count computes the gene copy count as the mean GC-corrected read depth for all informative locations in the gene, divided by the mean control read depth *μ*. To obtain the final copy count for each family, AmpliCoNE-count reports the total sum of the copy counts of all the genes in the family.

### Simulation-based validation of AmpliCoNE

To evaluate the accuracy of AmpliCoNE, we ran simulations. There are nine *TSPY* genes (six functional + three pseudogenes), six *RBMY* genes and two *VCY* genes in the hg38 reference. We added different copies of these three ampliconic gene families to the Y chromosome (S1 Table) to simulate reads. The total number of gene copies in the three custom references used to generate the simulated reads were 22/7/4 copies (for *TSPY/RBMY/VCY*, respectively) in set 1; 29/12/2 copies in set 2; 23/9/3 copies in set 3. Using wgsim [0.3.2] [76] we simulated 666 million paired-end reads of length 101 bp and insert size of 260 bp (the exact parameters were "-d 260 -N 666873346–1 101–2 101 -S 9 -e 0 -r 0 -R 0"). The reads from the three simulated datasets were aligned to the hg38 reference genome using BWA MEM[v0.7.15] [77]. The SAM files were sorted and PCR duplicates were removed using the PICARD toolkit [v1.128] [78]. Finally, samtools [v1.3.1] [79] were used to index the alignments. The sorted indexed BAM files were presented as input to AmpliCoNE-count.

### Datasets

We used mRNA sequencing data for 170 testis samples with matched whole-genome sequencing (WGS) data from the GTEx project [25]. The GTEx RNA-seq libraries were generated with the Illumina TruSeq protocol and whole-genome sequencing was performed with paired-end reads ranging from 100 bp to 150 bp in length with target insert size of 350–370 bp [25]. As the validation dataset for AmpliCoNE, we used WGS data from four males (depth of coverage ranging from ~45-50x in HG002 and HG003, ~300x in HG005 and ~100x in HG006) sequenced by the GIAB consortium [30].

### Pipeline for human WGS analysis

The Y-chromosome-specific alignments of the GTEx dataset were extracted from dbGAP using the SRA toolkit [80]. From the alignments, we extracted the reads and aligned them to the hg38 reference genome using bwa-mem [v0.7.15] [77]. The SAM files were sorted and PCR duplicates were removed using PICARD toolkit [v1.128] [78]. Finally, samtools [v1.3.1] [79] were used to index the read alignment files. The generated BAM files were presented as input to AmpliCoNE-count to estimate ampliconic copy number.

AMpliCoNE-build requires the locations of all the gene copies, in the reference genome, for each ampliconic gene family. While the locations of functional copies are already annotated in hg38, these do not include highly similar pseudogenized copies. These are necessary to include since they will affect the read mappings. For each family, we therefore took an arbitrary annotated copy of a gene, and used BLAT [81] to find all sites aligning with >99% identity (S13 Table). These locations were given as input to AmpliCoNE-build.

### Experimental validation with droplet digital PCR (ddPCR)

In order to validate the *in silico* ampliconic gene copy number count in four individuals sequenced by the GIAB consortium [30], we acquired their DNA (NA24385, NA24149, NA24631, and NA24694) from Coriell and performed ddPCR for all nine Y ampliconic gene families. In order to infer the copy number of these gene families we used *SRY*, a single-copy gene on the Y chromosome, and *RPP30*, a two-copy autosomal gene, as references. We ran ddPCR for each sample in triplicates using EvaGreen dsDNA dye (Bio-Rad) on the Biorad QX200 digital droplet platform with the protocol and primers from our previous publication [82]. The results were analyzed using QuantaSoft software. Subsequently, after removal of outliers, the concentration (copies/uL) of each ampliconic gene family of interest was divided by the concentration of the references, *SRY* and *RPP30* (S4 Table).

### Estimating gene expression levels

Gene expression estimates were obtained using the kallisto-DESeq2 pipeline described below. The standard human (hg38) RefSeq transcripts obtained from the UCSC Genome Browser [83] were used as reference. We generated an index for the reference using the kallisto [v0.43.0] index function with default parameters [84]. For each sample we obtained read counts per transcript using the kallisto quant (—bias,—seed = 9,—bootstrap-samples = 100) function. The hg38 refFlat file containing the transcript-to-gene mapping information was obtained from the UCSC Genome Browser [83] annotation database, which was used to convert the transcript-level read counts to gene-level expression levels using tximport package [v1.2.0] [85]. Since there were no replicates for the samples, we set the 170 sample ids as different conditions in the design, and the gene-level read counts for 170 RNA-seq samples were normalized using DESeq2 [v1.14.1] [86]. Additionally, read counts based on the vst (Variance Stabilizing Transformation) function in DESeq2 were used to check for outliers. To identify outliers in the dataset we performed Principal Component Analysis using the prcomp() function on the vst-based normalized read counts. When we plotted the first and second principal components, we found 21 samples outside the main cluster of the remaining 149 samples (S12 Fig). We followed steps described in DEseq2 vignettes and plotted the heatmap of sample-to-sample distance for the top 1,000 highly expressed genes to identify outliers visually and we found the same 21 samples as outliers. Thus, we filtered out these 21 samples and utilized the expression values for the nine ampliconic families in the remaining 149 samples in the downstream analysis. We summed the expression values for all the gene copies within a gene family to obtain family-level expression values.

### Human Y haplogroup determination

Yhaplo [v1.0.11] [87] was used to predict Y haplogroup of the samples. The version of Yhaplo[1.0.11] we used expects the SNP coordinates consistent with the hg19 [88] version of the human reference. The Y-chromosome-specific BAM files downloaded from dbGAP were aligned to the hg19 version of the human reference using BWA MEM. We directly converted the downloaded BAM files into pileup format using samtools mpileup function. A custom script was used to convert the pileup file into Yhaplo-compatible input format. We annotated the Y haplotype for all the samples in the dataset using Yhaplo default parameters.

### Code availability

Code used in the manuscript is available at github link: https://github.com/makovalab-psu/GTEx_Testis_Analysis. Steps to install and use AmpliCoNE are available at github: https://github.com/makovalab-psu/AmpliCoNE-tool

## Supporting information

S1 Table Copy number counts in the simulated sets. The numbers in the table represent the total number of gene copies for each ampliconic family present on the Y chromosome reference and used to simulate paired end fastq files and the copy number estimates using AmpliCoNE (Observed). The gene families in bold have custom copy numbers in each set.
(XLSX)

S2 Table The ampliconic gene copy number estimates across technical and biological replicates in three male samples from the GIAB consortium. Ashkenazim Son (HG002), Ashkenazim Father (HG004), and Chinese Father (HG006). The ampliconic gene copy number was estimated for two sequencing runs per individual which are represented as separate column in the table.
(XLSX)

S3 Table The ampliconic gene copy number estimates across different depths of coverage of Y chromosome in Ashkenazim Son (HG002 Run1) from the GIAB consortium. The reads were subsampled using samtools view function.
(XLSX)

S4 Table ddPCR-based ampliconic gene copy number estimates for four males from the GIAB consortium. Replicates a, b and c are the copy numbers from the three replicate experiments that were performed, and their mean value is used as the final estimate of the copy number. N/A—not available.
(XLSX)

S5 Table Sample IDs of the 170 GTEx samples used initially and retained after filtering for outliers in the gene expression analysis. (XLSX)

S6 Table Median, standard deviation (SD) and range of copy number (CN, N = 167) and gene expression (GE) values per ampliconic gene family (N = 149). (XLSX)

S7 Table Copy number and gene expression correlation values. Spearman correlation coefficient values (r) and P-values for each gene family are calculated using cor.test() function in R. The P-values cutoff after Bonferroni correction for nine tests is 0.05/9 ≈ 0.006).
(XLSX)

S8 Table P-values from permutation tests for copy number differences between haplogroup pairs. Given two haplogroups, to test whether the difference in copy number between the haplogroups is significant, we compared the true difference in mean copy number between haplogroups to the difference in mean of 1 million random permutations (randomly rearranged the haplogroup assignment of the two haplogroups). The *P*-value represents how many permuted mean-differences are larger than the one we observed in our actual data. P-values that pass a Bonferroni corrected cutoff for 54 tests (0.05/54 = 0.00093) are highlighted in bold.
(XLSX)

S9 Table P-values from permutation test for gene expression differences between haplogroup pairs. Given two haplogroups, to test if the difference in gene expression between the haplogroups is significant or not, we compared the true difference in mean gene expression between haplogroups to the difference in mean of 1 million (M) random permutations (randomly rearranged the haplogroup assignment). The *P*-value represents how many permuted mean-differences are larger than the one we observed in our actual data. None of the *P*-values pass a Bonferroni corrected cutoff for fifty four tests (0.05/54 = 0.00093).
(XLSX)

S10 Table Correlation between gene expression and age. Spearman correlation coefficient values (r) and P-values for each of the gene family are calculated using cor.test() function in R. P-values that pass a Bonferroni corrected cutoff for nine tests (0.05/9 ≈0.006) are highlighted in bold.
(XLSX)

S11 Table Ampliconic gene homologs and their expression patterns. The homologs of ampliconic genes were obtained from a recent review. The expression pattern is obtained from the human protein atlas (HPA). Tissue-enriched: expression in one tissue is at least five-fold higher than that in all other tissues/cell lines. Tissue-enhanced (five-fold higher average transcripts per million (TPM) in one or more tissues/cell lines compared to the mean TPM of all tissues/cell lines). Ubiquitous (≥ 1 TPM in all tissues/cell lines). Mixed (detected in at least one tissue/cell line and in none of the above categories).
(XLSX)

S12 Table Predicted function of ampliconic gene families. The table was adapted from Table 1 of Paulo Navarro-Costa’s review on ampliconic gene families. All the gene families are linked to spermatogenesis and infertility.
(XLSX)

S13 Table Numbers of high identity alignments to the references for each Y ampliconic gene family. BLAT was used to find all sites with >99% identity. For *TSPY* there are three pseudogene copies in the reference which share identity with parts of functional copies of *TSPY* genes.
(XLSX)

S1 Fig Variation in copy number of ampliconic gene families. In the dotplot the X-axis represents natural log of median copy number and the Y-axis is the natural log of variance in copy number for the 167 individuals analyzed. The blue line represents the linear regression fit (median ~ var) with an R^2^ value of 0.91. The color of each dot is labeled with ampliconic gene family described in the legend.
(TIF)

S2 Fig Variation in gene expression of ampliconic gene families. In the dotplot, the X-axis represents natural log of the median normalized gene expression values and the Y-axis represents natural log of the variance in gene expression for the 149 individuals analyzed. The blue line represents the linear regression fit (median ~ var) with an R^2^ value of 0.99. The color of each dot is labeled with ampliconic gene family described in the legend.
(TIF)

S3 Fig The relationship between expression level and copy number (N = 149). Within each scatter plot, the X-axis represents copy number values and Y-axis represents the normalized gene expression values. The Spearman correlations were calculated using the cor.test() function in R and the *P*-values are in brackets. The gray line represents the linear function fitted to the given data points. The nine scatter plots represent the relationship between expression and copy number for each of the nine ampliconic gene families. There is no significant relationship in either of the nine gene families (Bonferroni correction p-value cutoff of 0.05/9 = 0.006).
(TIF)

S4 Fig The relationship between gene expression and copy number for individuals with an R1b (European) subhaplogroup (N = 77). Within each scatter plot, the X-axis represents the copy number values and Y-axis represents the normalized gene expression values. The Spearman correlations were calculated using the cor.test() function in R and the *P*-values are shown in brackets. The gray line represents the linear function fitted to the given data points. The nine scatter plots represent the relationship between expression and copy number of the ampliconic gene families. There is no significant relationship in either of the nine gene families (Bonferroni correction p-value cutoff of 0.05/9 = 0.006).
(TIF)

S5 Fig The relationship between gene expression and copy number for individuals with a I1a (European) subhaplogroup (N = 15). Within each scatter plot the X-axis represents the copy number values and Y-axis represents the normalized gene expression values. The Spearman correlations were calculated using the cor.test() function in R and the P-values are shown in brackets. The gray line represents the linear function fitted to the given data points. The nine scatter plots represent the relationship between expression and copy number of the ampliconic gene families. There is no significant relationship in either of the nine gene families (Bonferroni correction p-value cutoff of 0.05/9 = 0.006).
(TIF)

S6 Fig The relationship between gene expression and copy number for individuals with a E1b (African) subhaplogroup (N = 22). Within each scatter plot the X-axis represents the copy number values and Y-axis represents the normalized gene expression values. The Spearman correlations were calculated using the cor.test() function in R and the P-values are shown in brackets. The gray line represents the linear function fitted to the given data points. The nine scatter plots represent the relationship between expression and copy number of the ampliconic gene families. There is no significant relationship in either of the nine gene families (Bonferroni correction p-value cutoff of 0.05/9 = 0.006).
(TIF)

S7 Fig The relationship between gene expression and age in the individuals analyzed (N = 149). The nine scatterplots represent the nine ampliconic gene families with their names as the title of their respective plot. Within each scatter plot the Y-axis represents the age and X-axis represents the gene expression values. The Spearman correlations were calculated using the cor.test() function in R and the P-values are shown in brackets. There is no significant relationship between age and expression in all the nine families (Bonferroni correction p-value cutoff of 0.05/9 = 0.006). The gray line represents the linear function fitted to the points in the plot.
(TIF)

S8 Fig The relationship between gene expression and age for individuals with a R1b (European) subhaplogroup (N = 77). The nine scatterplots represent the nine ampliconic gene families with their names as the title of their respective plot. Within each scatter plot the Y-axis represents the age and X-axis represents the gene expression values. The Spearman correlations were calculated using the cor.test() function in R and the P-values are shown in brackets. There is no significant relationship between age and expression in all the nine families (Bonferroni correction p-value cutoff of 0.05/9 = 0.006). The gray line represents the linear function fitted to the points in the plot.
(TIF)

S9 Fig The relationship between gene expression and age for individuals with a I1a (European) haplogroup (N = 15). The nine scatterplots represent the nine ampliconic gene families with their names as the title of their respective plot. Within each scatter plot the Y-axis represents the age and X-axis represents the gene expression values. The Spearman correlations were calculated using the cor.test() function in R and the P-values are shown in brackets. There is no significant relationship between age and expression in all the nine families (Bonferroni correction p-value cutoff of 0.05/9 = 0.006). The gray line represents the linear function fitted to the points in the plot.
(TIF)

S10 Fig The relationship between gene expression and age for individuals with a E1b (African) haplogroup (N = 22). The nine scatterplots represent the nine ampliconic gene families with their names as the title of their respective plot. Within each scatter plot the Y-axis represents the age and X-axis represents the gene expression values. The Spearman correlations were calculated using the cor.test() function in R and the P-values are shown in brackets. There is significant relationship between age and expression in *HSFY* and *PRY* families (Bonferroni correction p-value cutoff of 0.05/9 = 0.006). The gray line represents the linear function fitted to the points in the plot.
(TIF)

S11 Fig Combination of expression level differences and individual-level relationship between Y ampliconic gene families and their non-Y homologs can better explain the possible scenarios of evolution for the former. Within each row (A-D), the plot on the left represents the expression level differences between Y ampliconic genes (blue boxplot) and their non-Y homologs (orange boxplot), the plot in the middle represents the individual level relationship between Y ampliconic genes (X-axis) and their non-Y homologs (Y-axis) and on the right are the expected scenarios of evolution. Assuming non-Y homologs represent ancestral expression levels, higher expression of Y ampliconic genes implies independent expression (A, B) and lower expression implies dosage regulation (C, D). Negative correlation among ampliconic genes and their non-Y homologs suggests lack of co-regulation (B, D) and a positive correlation suggests coregulation of gene expression(A, C).
(TIF)

S12 Fig PCA plot of all 170 samples using Variance Stabilizing Transformation (VST) normalized read counts. All the points with greater than 20 PC1 value (X-axis) were filtered out.
(TIF)
