# Supplementary figures and images for: Dosage regulation, and variation in gene expression and copy number of human Y chromosome ampliconic genes
Source: PLoS Genet. 2019 Sep 16;15(9):e1008369. doi: 10.1371/journal.pgen.1008369 (PMC6772104; doi:10.1371/journal.pgen.1008369)

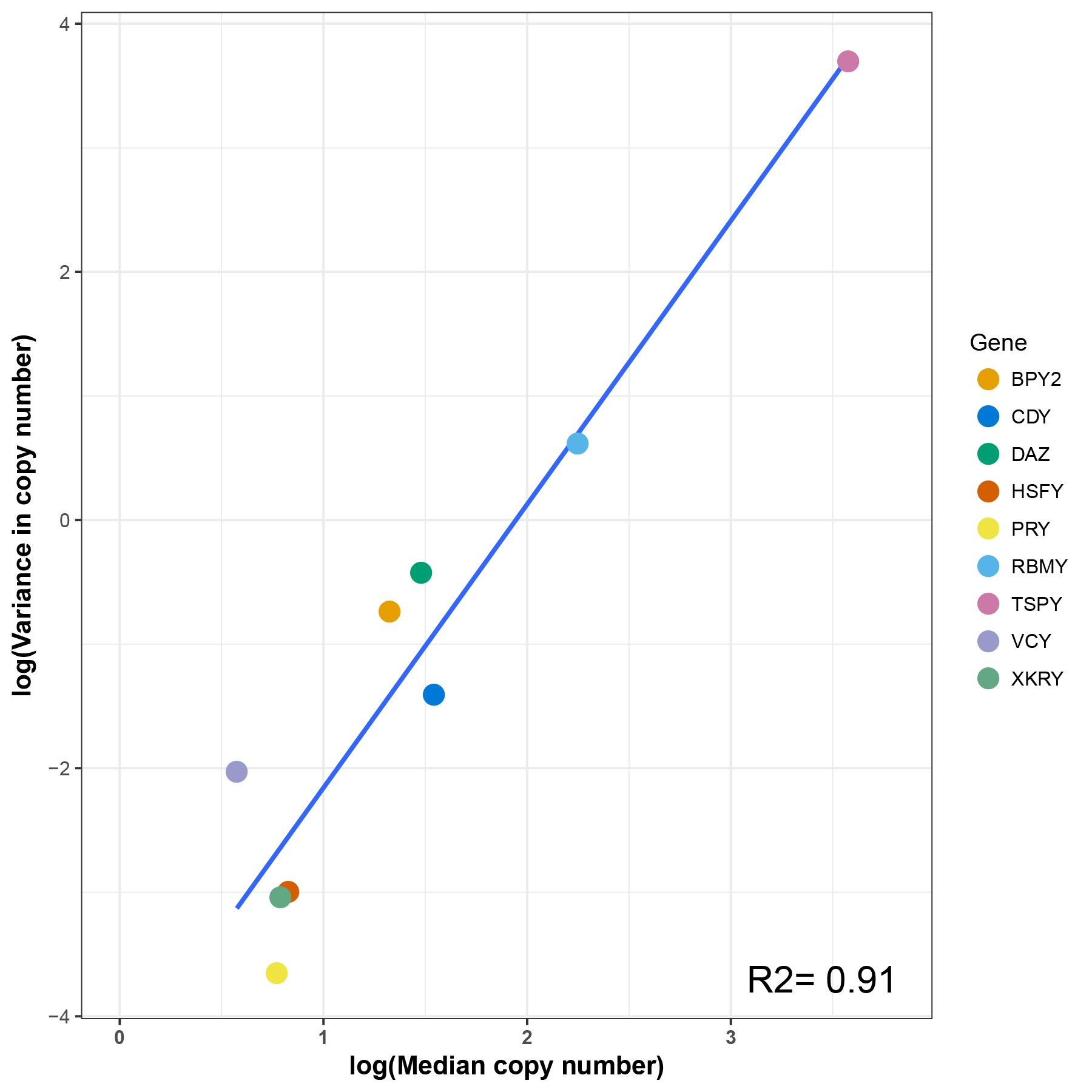

Supplement: S1 Fig — In the dotplot the X-axis represents natural log of median copy number and the Y-axis is the natural log of variance in copy number for the 167 individuals analyzed. The blue line represents the linear regression fit (median ~ var) with an R2 value of 0.91. The color of each dot is labeled with ampliconic gene family described in the legend. (TIF) [file pgen.1008369.s014.tif]

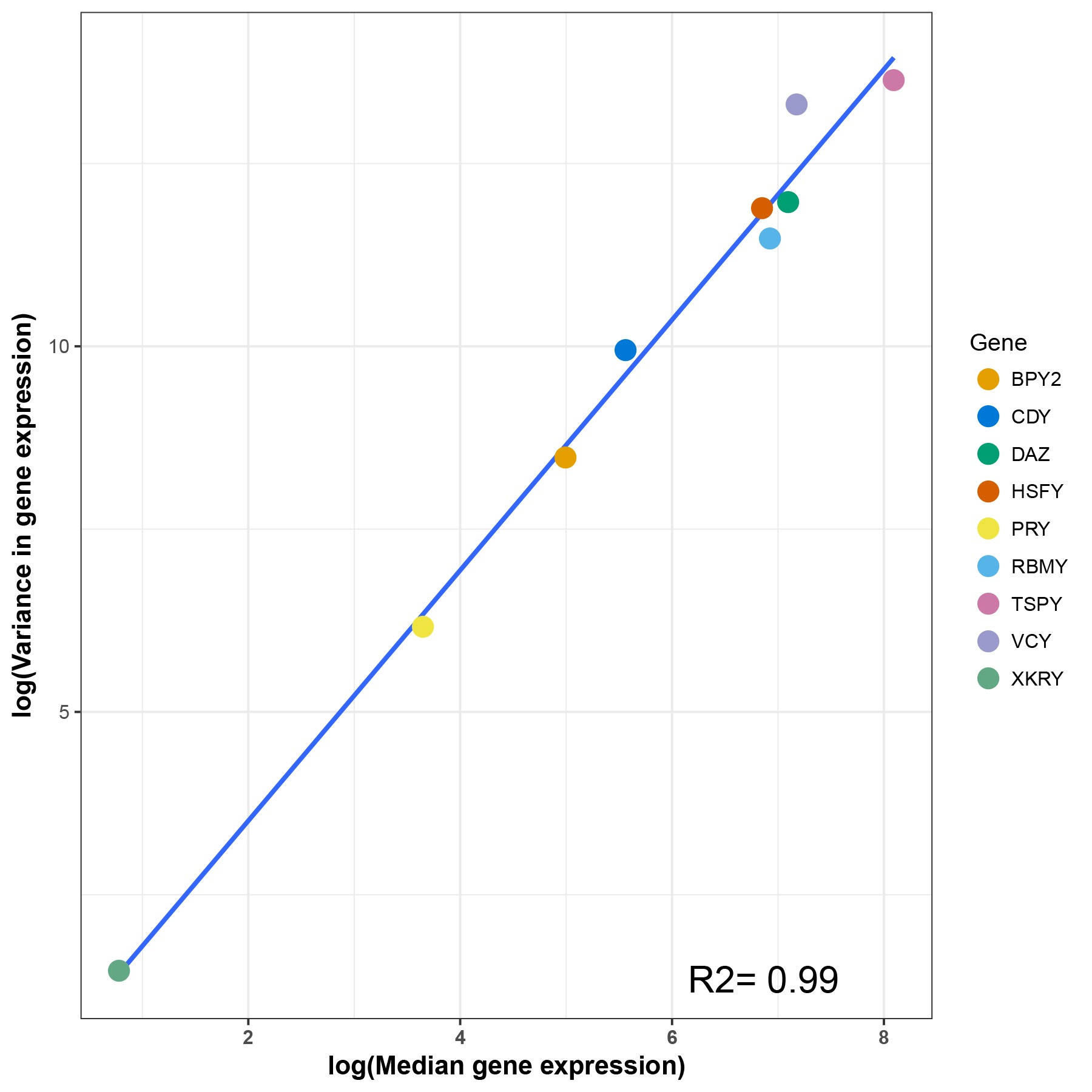

Supplement: S2 Fig — In the dotplot, the X-axis represents natural log of the median normalized gene expression values and the Y-axis represents natural log of the variance in gene expression for the 149 individuals analyzed. The blue line represents the linear regression fit (median ~ var) with an R2 value of 0.99. The color of each dot is labeled with ampliconic gene family described in the legend. (TIF) [file pgen.1008369.s015.tif]

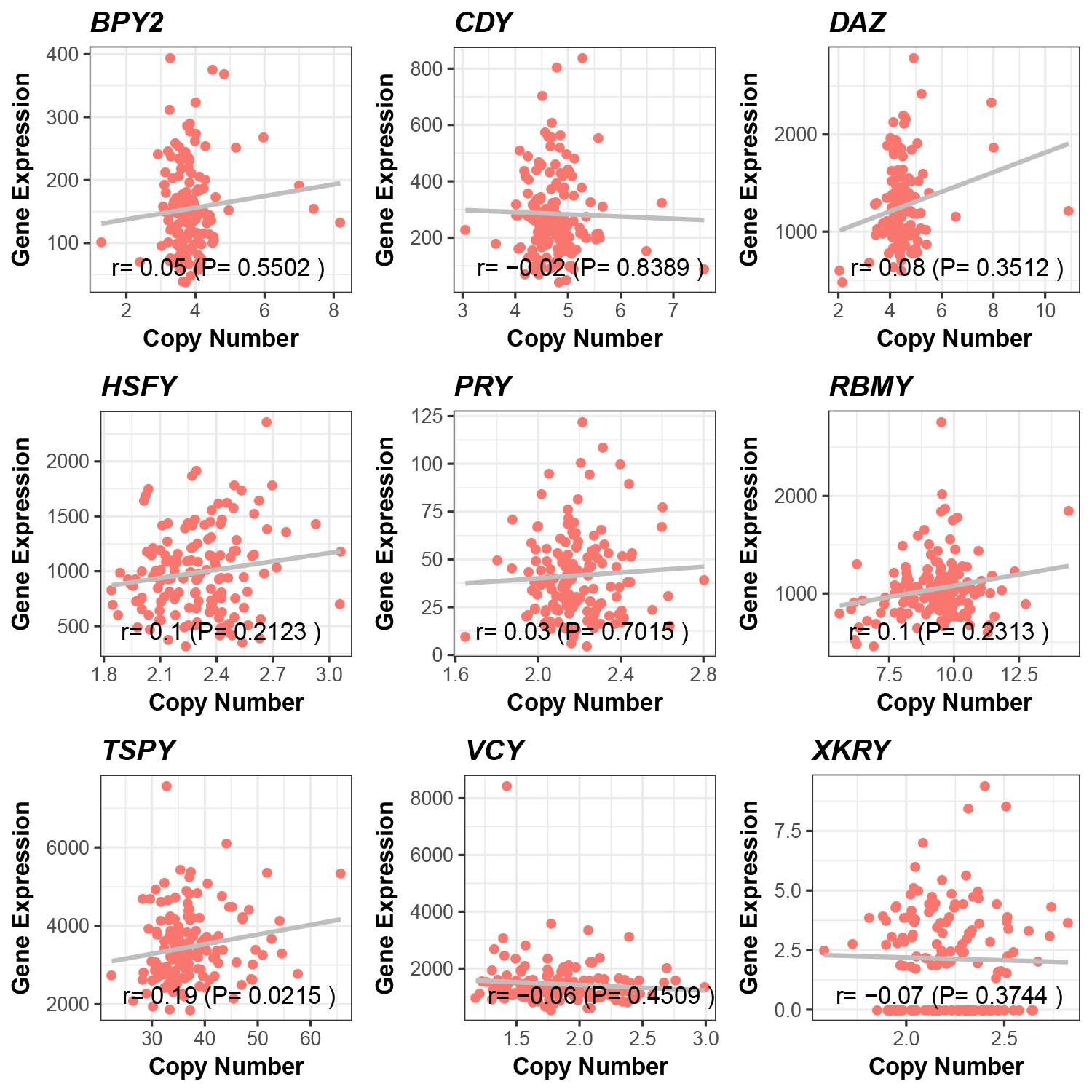

Supplement: S3 Fig — Within each scatter plot, the X-axis represents copy number values and Y-axis represents the normalized gene expression values. The Spearman correlations were calculated using the cor.test() function in R and the P-values are in brackets. The gray line represents the linear function fitted to the given data points. The nine scatter plots represent the relationship between expression and copy number for each of the nine ampliconic gene families. There is no significant relationship in either of the nine gene families (Bonferroni correction p-value cutoff of 0.05/9 = 0.006). (TIF) [file pgen.1008369.s016.tif]

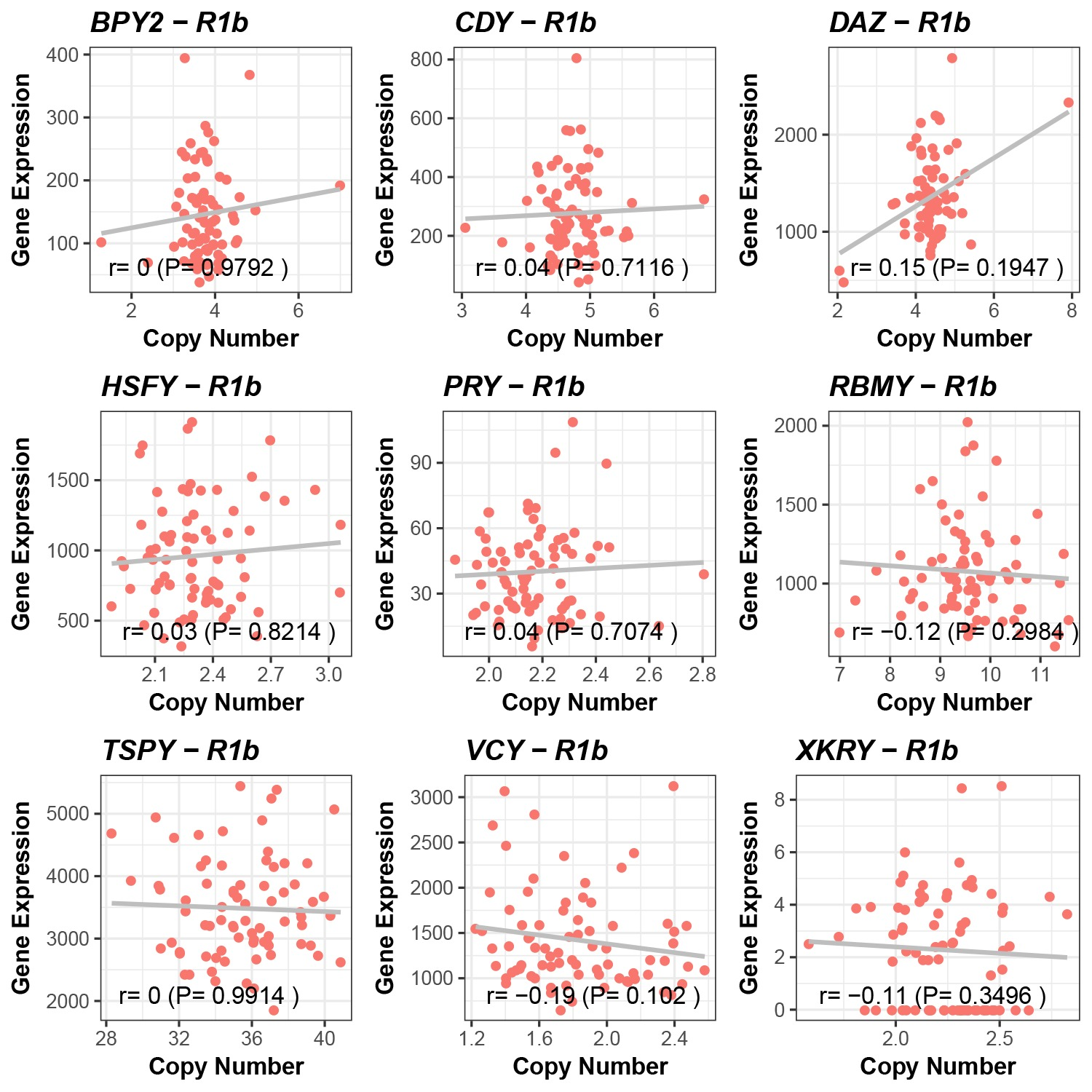

Supplement: S4 Fig — Within each scatter plot, the X-axis represents the copy number values and Y-axis represents the normalized gene expression values. The Spearman correlations were calculated using the cor.test() function in R and the P-values are shown in brackets. The gray line represents the linear function fitted to the given data points. The nine scatter plots represent the relationship between expression and copy number of the ampliconic gene families. There is no significant relationship in either of the nine gene families (Bonferroni correction p-value cutoff of 0.05/9 = 0.006). (TIF) [file pgen.1008369.s017.tif]

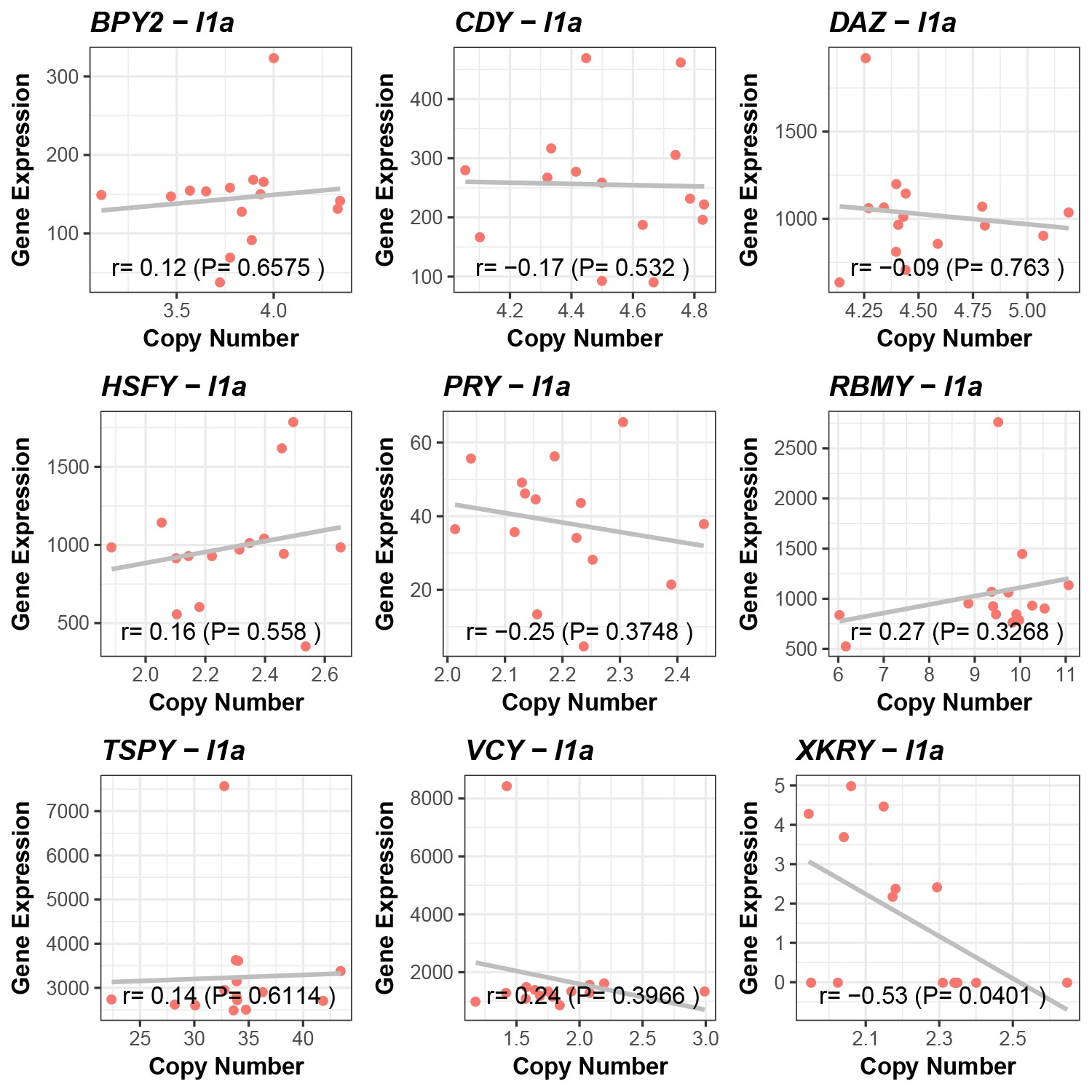

Supplement: S5 Fig — Within each scatter plot the X-axis represents the copy number values and Y-axis represents the normalized gene expression values. The Spearman correlations were calculated using the cor.test() function in R and the P-values are shown in brackets. The gray line represents the linear function fitted to the given data points. The nine scatter plots represent the relationship between expression and copy number of the ampliconic gene families. There is no significant relationship in either of the nine gene families (Bonferroni correction p-value cutoff of 0.05/9 = 0.006). (TIF) [file pgen.1008369.s018.tif]

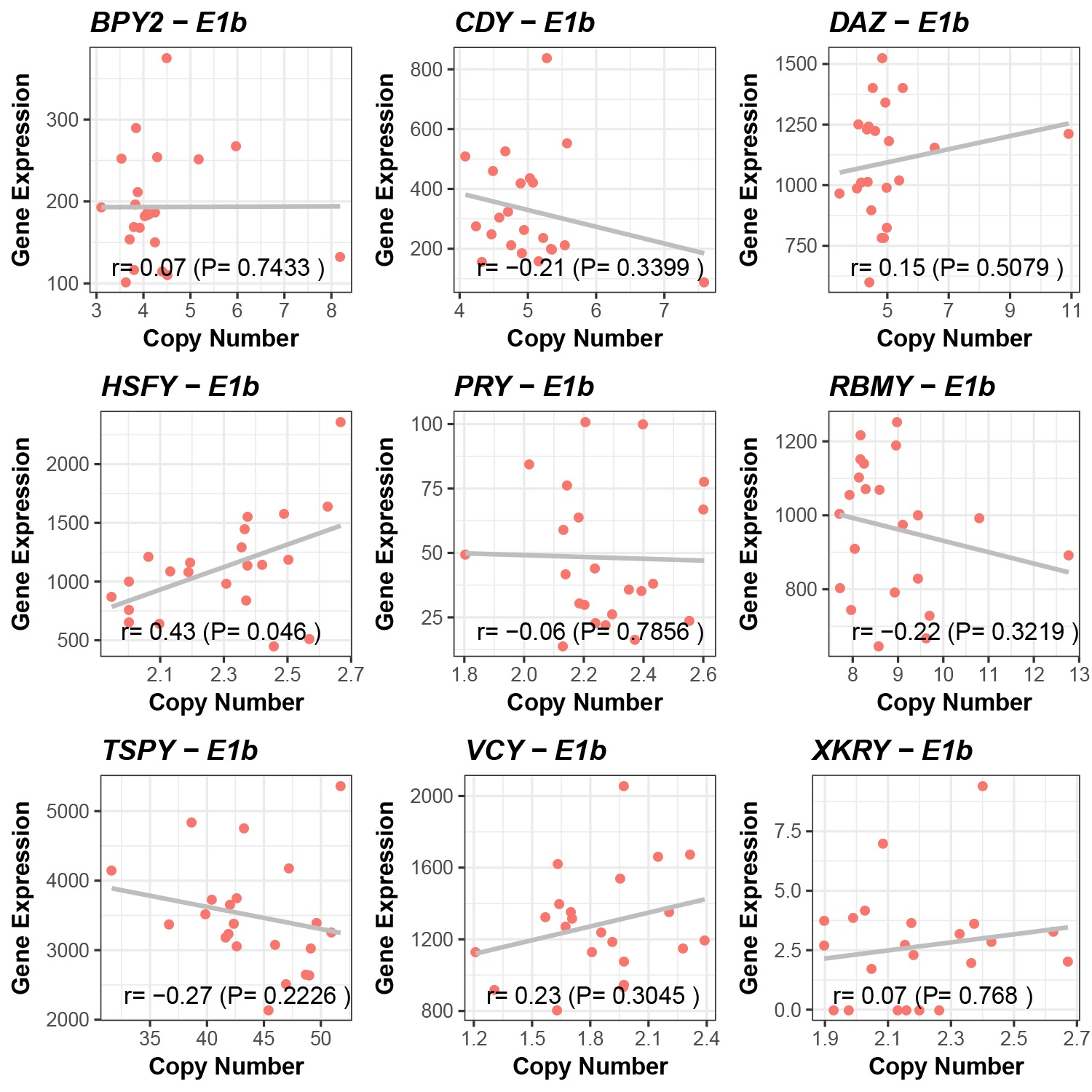

Supplement: S6 Fig — Within each scatter plot the X-axis represents the copy number values and Y-axis represents the normalized gene expression values. The Spearman correlations were calculated using the cor.test() function in R and the P-values are shown in brackets. The gray line represents the linear function fitted to the given data points. The nine scatter plots represent the relationship between expression and copy number of the ampliconic gene families. There is no significant relationship in either of the nine gene families (Bonferroni correction p-value cutoff of 0.05/9 = 0.006). (TIF) [file pgen.1008369.s019.tif]

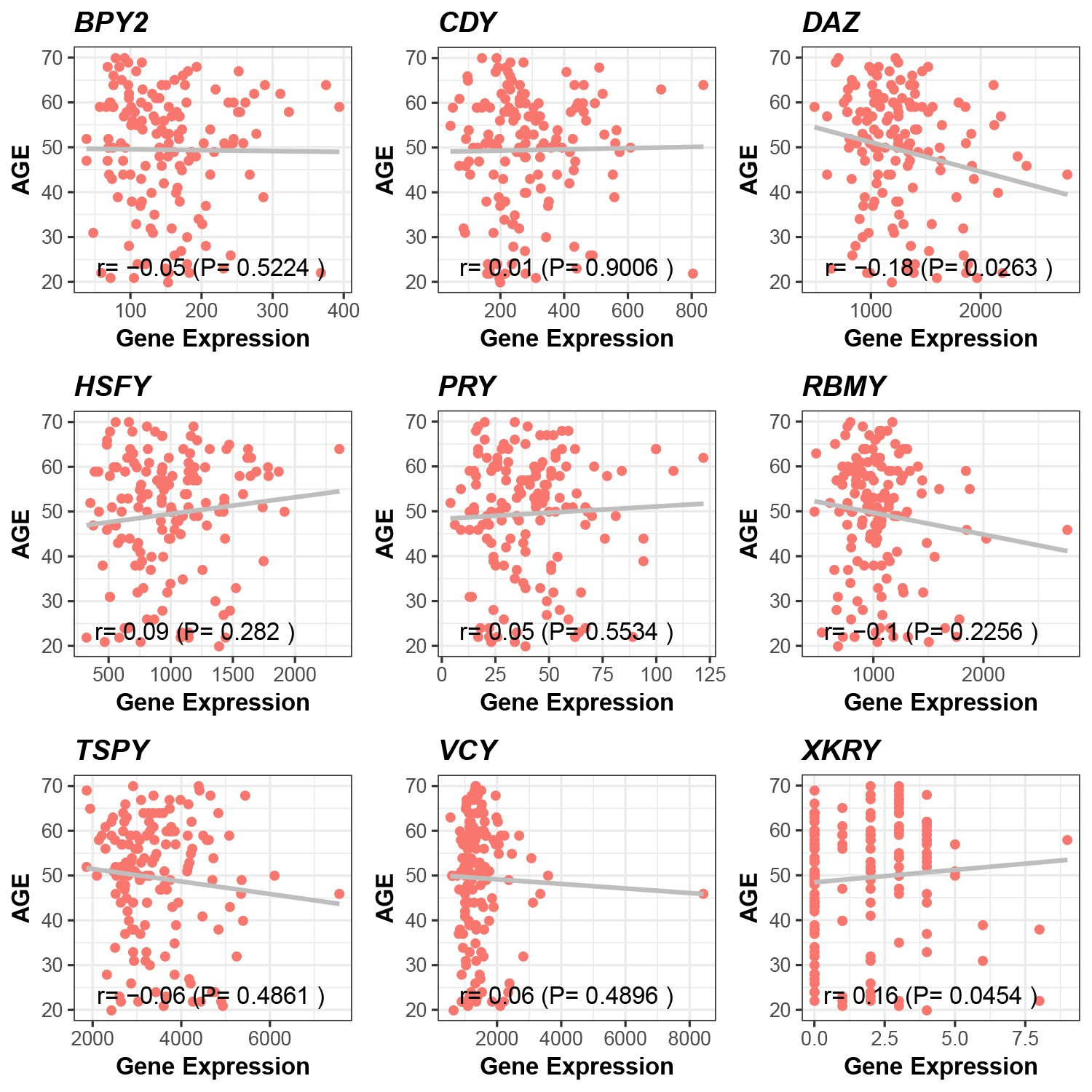

Supplement: S7 Fig — The nine scatterplots represent the nine ampliconic gene families with their names as the title of their respective plot. Within each scatter plot the Y-axis represents the age and X-axis represents the gene expression values. The Spearman correlations were calculated using the cor.test() function in R and the P-values are shown in brackets. There is no significant relationship between age and expression in all the nine families (Bonferroni correction p-value cutoff of 0.05/9 = 0.006). The gray line represents the linear function fitted to the points in the plot. (TIF) [file pgen.1008369.s020.tif]

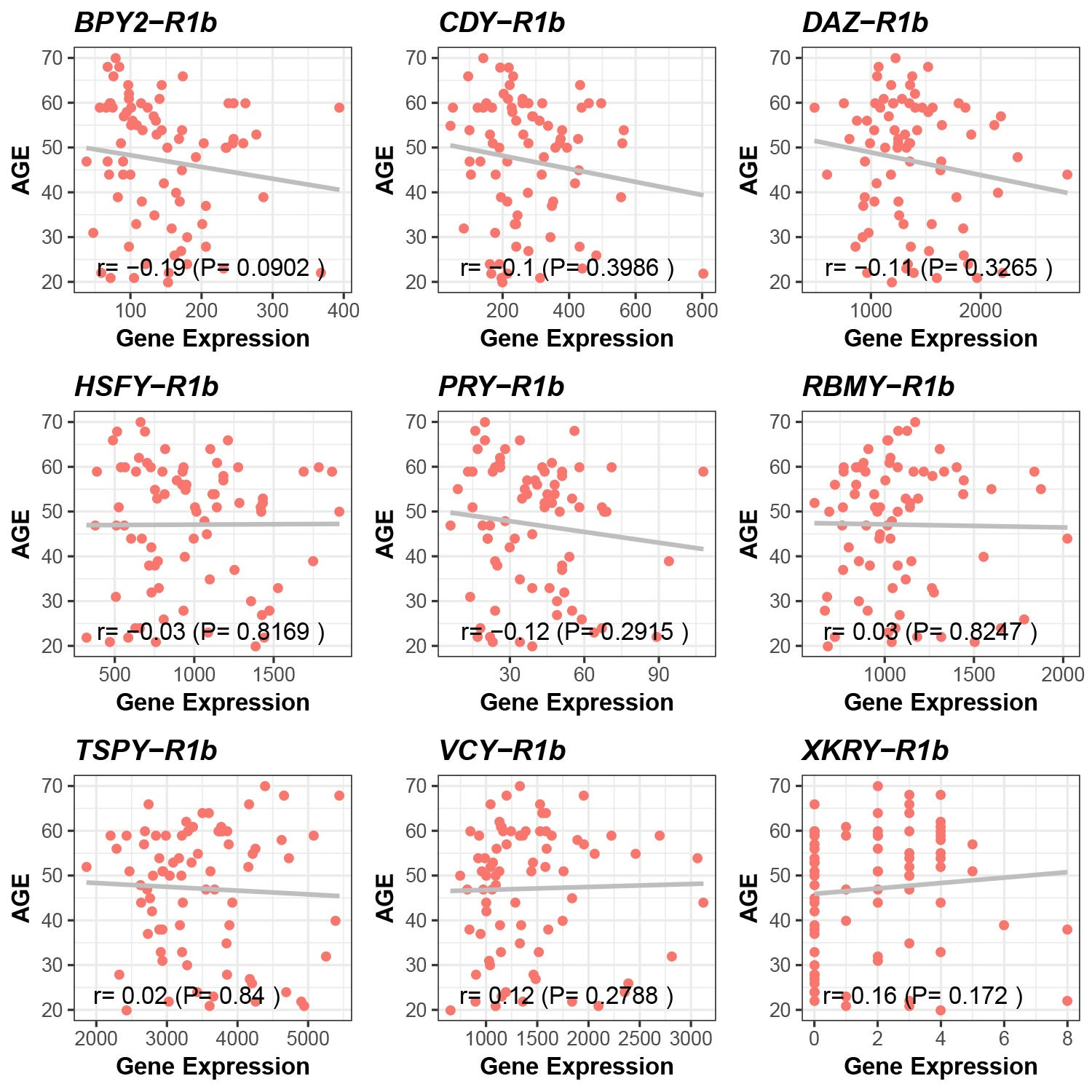

Supplement: S8 Fig — The nine scatterplots represent the nine ampliconic gene families with their names as the title of their respective plot. Within each scatter plot the Y-axis represents the age and X-axis represents the gene expression values. The Spearman correlations were calculated using the cor.test() function in R and the P-values are shown in brackets. There is no significant relationship between age and expression in all the nine families (Bonferroni correction p-value cutoff of 0.05/9 = 0.006). The gray line represents the linear function fitted to the points in the plot. (TIF) [file pgen.1008369.s021.tif]

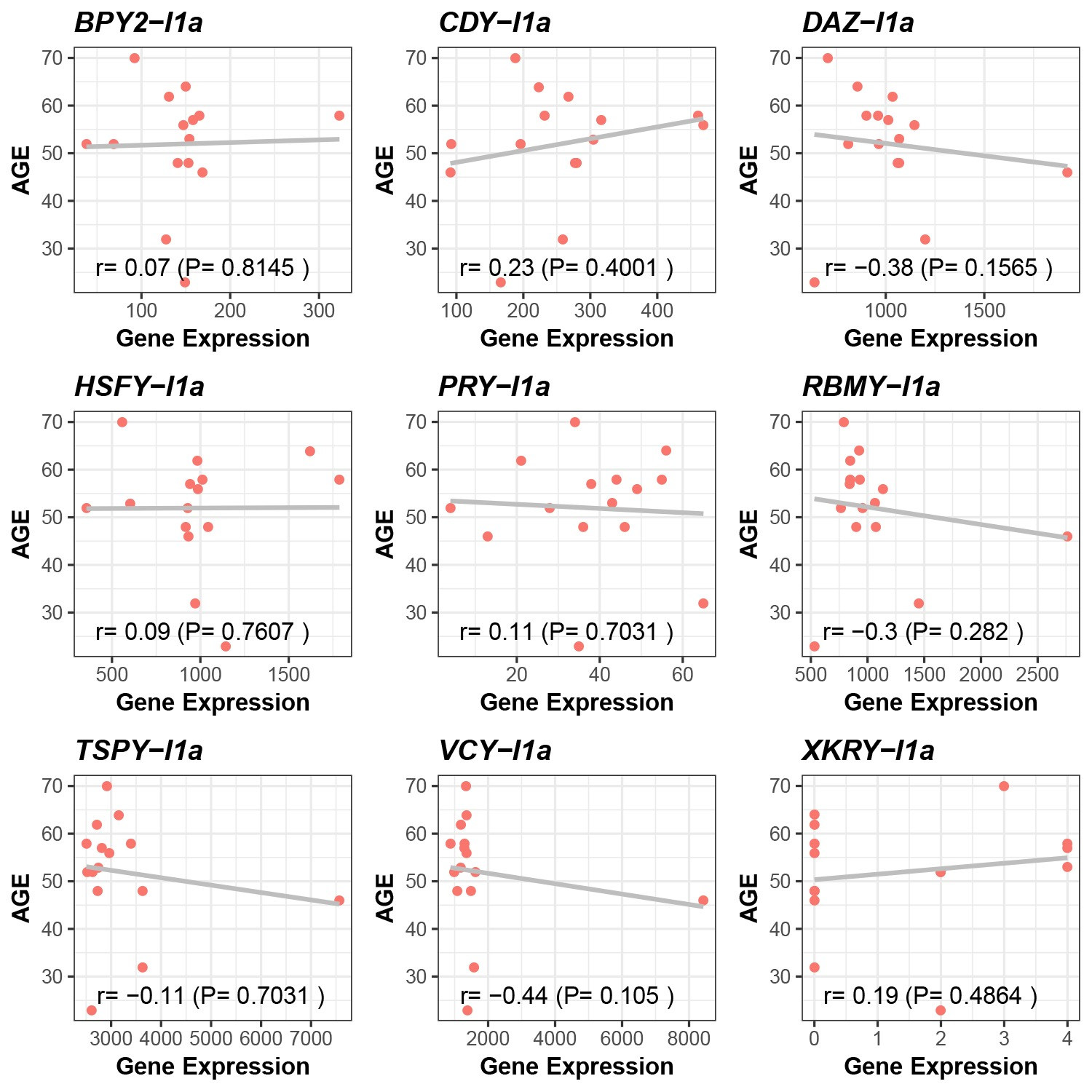

Supplement: S9 Fig — The nine scatterplots represent the nine ampliconic gene families with their names as the title of their respective plot. Within each scatter plot the Y-axis represents the age and X-axis represents the gene expression values. The Spearman correlations were calculated using the cor.test() function in R and the P-values are shown in brackets. There is no significant relationship between age and expression in all the nine families (Bonferroni correction p-value cutoff of 0.05/9 = 0.006). The gray line represents the linear function fitted to the points in the plot. (TIF) [file pgen.1008369.s022.tif]

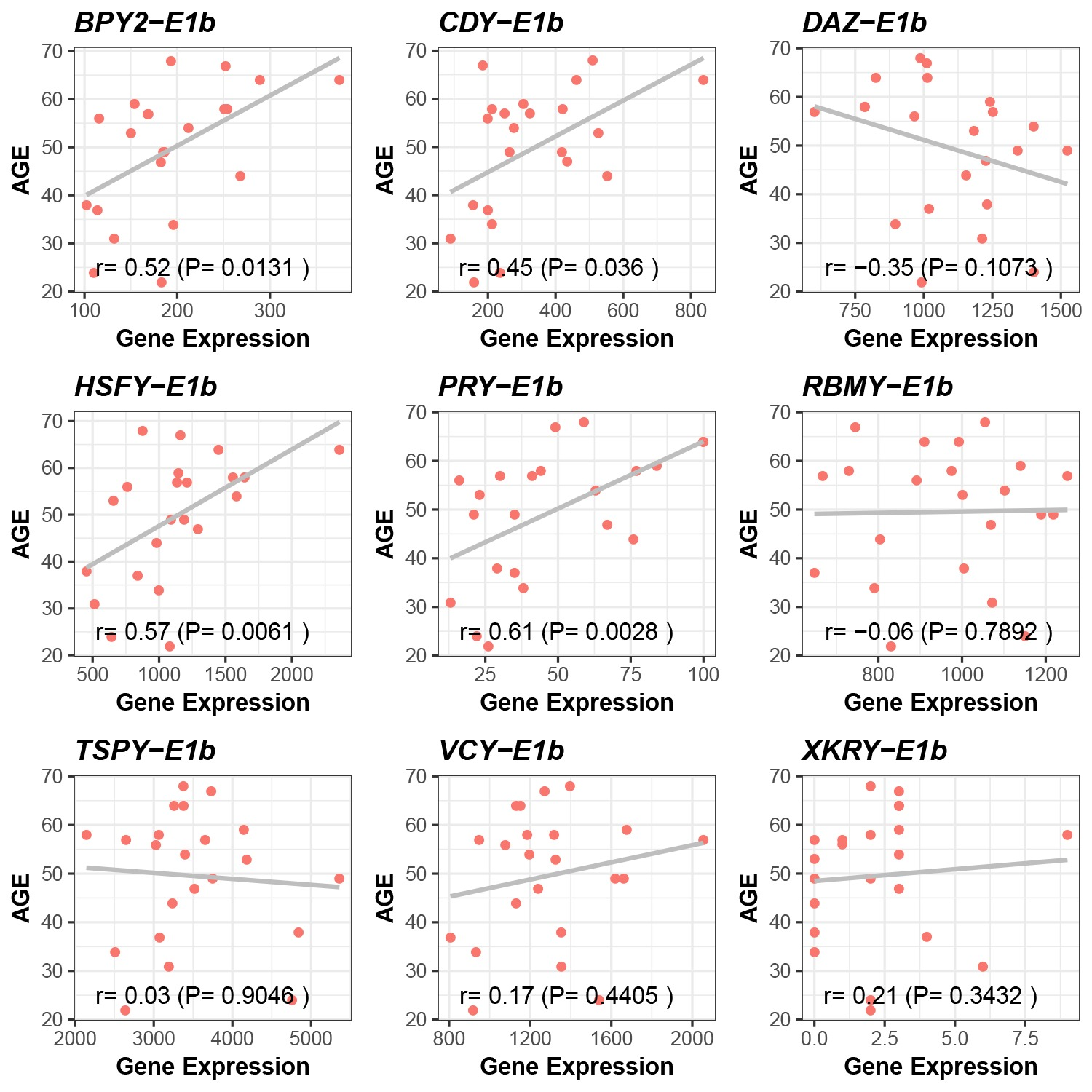

Supplement: S10 Fig — The nine scatterplots represent the nine ampliconic gene families with their names as the title of their respective plot. Within each scatter plot the Y-axis represents the age and X-axis represents the gene expression values. The Spearman correlations were calculated using the cor.test() function in R and the P-values are shown in brackets. There is significant relationship between age and expression in HSFY and PRY families (Bonferroni correction p-value cutoff of 0.05/9 = 0.006). The gray line represents the linear function fitted to the points in the plot. (TIF) [file pgen.1008369.s023.tif]

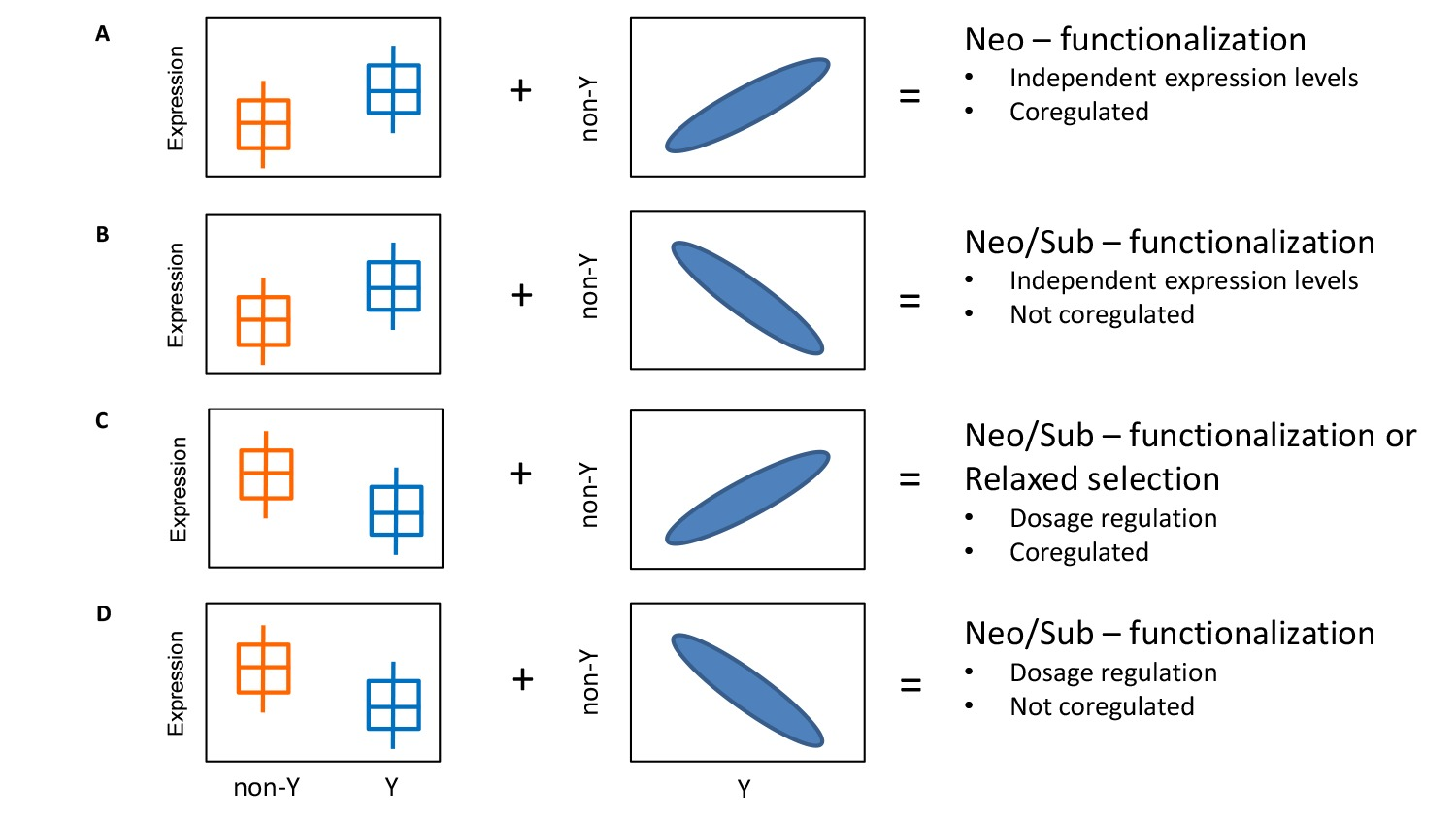

Supplement: S11 Fig — Within each row (A-D), the plot on the left represents the expression level differences between Y ampliconic genes (blue boxplot) and their non-Y homologs (orange boxplot), the plot in the middle represents the individual level relationship between Y ampliconic genes (X-axis) and their non-Y homologs (Y-axis) and on the right are the expected scenarios of evolution. Assuming non-Y homologs represent ancestral expression levels, higher expression of Y ampliconic genes implies independent expression (A, B) and lower expression implies dosage regulation (C, D). Negative correlation among ampliconic genes and their non-Y homologs suggests lack of co-regulation (B, D) and a positive correlation suggests coregulation of gene expression(A, C). (TIF) [file pgen.1008369.s024.tif]

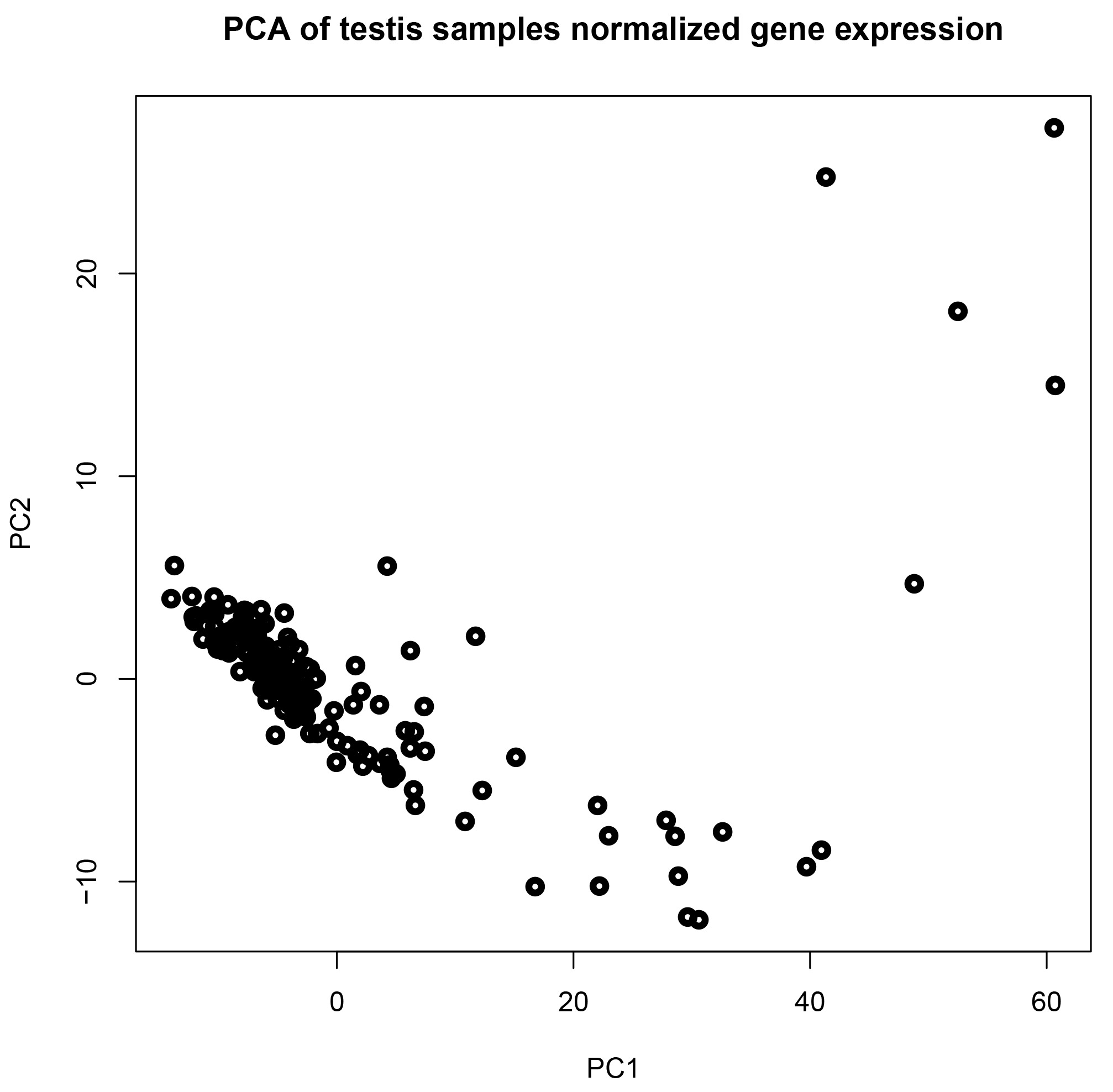

Supplement: S12 Fig — All the points with greater than 20 PC1 value (X-axis) were filtered out. (TIF) [file pgen.1008369.s025.tif]
